# Veterinary teaching in COVID-19 times: perspectives of university teaching staff

**DOI:** 10.3389/fvets.2024.1386978

**Published:** 2024-06-27

**Authors:** Miriam Kanwischer, Andrea Tipold, Elisabeth Schaper

**Affiliations:** ^1^Center for E-Learning, Didactics and Educational Research, University of Veterinary Medicine Hannover, Foundation, Hannover, Germany; ^2^Department of Small Animal Medicine and Surgery, University of Veterinary Medicine Hannover, Foundation, Hannover, Germany

**Keywords:** veterinary education, COVID-19 pandemic, e-learning, blended learning, didactic concept, digital education

## Abstract

The digitalization of university teaching has been taking place for many years and, in addition to traditional teaching formats such as practicals and face-to-face lectures, more and more e-learning courses have been used in veterinary education for several years. In the context of the COVID-19 pandemic, universities across Germany had to switch to an *ad-hoc*, purely digital summer semester. This study evaluated the experiences and implementation of the digital summer semester 2020 at the University of Veterinary Medicine Hannover (TiHo) Foundation from the perspective of the teaching staff. In addition to the technical equipment used by lecturers, this survey also focused on the effects of the digital semester on teaching and the future practicality of digital teaching formats and strategies in veterinary education. Therefore, a questionnaire was designed and distributed among lecturers involved in the digital summer semester 2020. One hundred and three completed questionnaires were evaluated. The results of the evaluation show that teachers see huge potential in blended learning as a teaching method in veterinary education. In addition, teachers were able to digitize teaching well with the available hardware and software. The teaching staff saw difficulties above all in the loss of practical training and in the digitalization of practical exercises. Teachers also needed significantly more time to plan and implement digital teaching compared to pure face-to-face teaching. In summary blended learning offers many advantages, such as increased flexibility for students and teaching staff. In order to be able to use digital teaching methods and strategies profitably in veterinary education in the future, well thought-out didactic concepts and further technical expansion of the universities are required. In addition, the digital skills of teaching staff should be further trained and promoted.

## 1 Introduction

The COVID-19 pandemic spread worldwide at the beginning of 2020 and led to an *ad hoc* changeover of the summer semester from face-to-face to purely digital online teaching throughout Germany from mid-March 2020 (1). At the University of Veterinary Medicine Hannover, Foundation (TiHo), Hannover, Germany, new learning and communication platforms such as Moodle and Microsoft® Teams were introduced and e-learning formats already tried and tested in veterinary medicine, such as “virtual patients” in CASUS® (2, 3), talks and lecture recordings, and educational videos (4, 5) as well as game-based learning with the Actionbound app (6) were further expanded and promoted. However, these could not be used as supplementary additional offerings as before, taking into account the requirements of individual teaching formats, but were *ad hoc* the main component of teaching in the digital summer semester 2020 at the TiHo. It became apparent that due to the short-term nature of the changeover, lecturers initially focused on the simplest and most direct 1:1 conversion of their lecture material and preferred to use digital tools that they had already used beforehand or that were easy to implement and required little preparation and planning time (7). Especially at the beginning of the summer semester, this led to an often purely synchronous or asynchronous teaching concept of the lecturers, which was mostly dominated by video conferences, lecture recordings and teaching videos (7, 8).

One particular task was the implementation of the comprehensive acquisition of practical skills in the training of veterinary students, which is regulated in Germany by the Veterinary Licensing Ordinance (9) and throughout Europe by Directive 2005/36/EC (10) and is further detailed in the European System of Evaluation of Veterinary Training (ESEVT) Standard Operating Procedure (SOP) (11). The directive and the Veterinary Licensing Ordinance have an impact on the entire practical training of veterinary medicine, which includes basic subjects such as chemistry and medical physics as well as specific veterinary subjects such as basic sciences, clinical sciences, animal production and food safety and quality, veterinary public health and one health concept.

The aim of this study was to investigate the implementation of veterinary teaching in the summer semester 2020 at the University of Veterinary Medicine Hannover (TiHo) from the lecturers’ perspective. Furthermore, the needs for digital teaching were to be recorded and it was to be investigated which digital teaching formats are becoming more relevant for modern veterinary teaching and should be used and promoted sustainably at the TiHo.

Against this background, the hypothesis that lecturers want to retain digital formats in the future was tested.

## 2 Materials and methods

To evaluate digital teaching in the summer semester 2020, lecturers were surveyed using an online questionnaire. This was created in LimeSurvey (LimeSurvey GmbH, Hamburg, Germany). Before the final implementation, the questionnaire was formally checked in terms of content, structure, internal logic, and duration by means of a pre-test. For this purpose, the questionnaire was sent in advance to 12 selected persons from the Center for E-Learning, Didactics and Training Research at the TiHo. After evaluating and implementing the results of the pre-test, the questionnaire was then adapted and finalized. The link to the questionnaire was sent to the TiHo lecturers’ email distribution lists in July 2020. The lecturers had a total of four weeks to answer the questionnaire, during which they were repeatedly reminded to do so by e-mail.

To collect the data, the questionnaire mainly included closed questions with one-best-answer questions as well as a few selected questions with multiple-select options. The majority of the questions were statements that the teaching staff were asked to rate using a four or five-point rating scale, as well as simple yes/no questions. In addition, four open-ended free text questions were also included in the questionnaire, which were then quantified using a qualitative content analysis.

The evaluation was divided into a total of 10 groups of questions, the results of which are presented from the following subject areas due to their scope:

- General personal details.
- Start of the digital semester.
- Technical requirements.
- Teaching in the digital semester.
- Effects of the digital semester on teaching.
- Outlook and general suggestions.

An excerpt of the questionnaire with the questions evaluated in the current publication was included in the Supplementary material. Further relevant results from other question groups in the survey will be published as part of the dissertation (12).

The descriptive analysis of the data was carried out using the spreadsheet program Microsoft® Office Excel 2013 (Microsoft Corporation, Redmond, WA, USA). The evaluation of the free text questions was carried out using qualitative content analysis. Further statistical analyses were carried out using the SAS Enterprise Guide Version 7.1 program (SAS Institute Inc., Cary, USA).

The statistical analysis of the data to determine correlations between different categorical variables was performed by creating contingency tables and an evaluation using Cramer’s V-value. For this purpose, the variables to be analyzed were first grouped together. The answer option “no response” was not included, so that the number of teachers per question may differ from the descriptive evaluation. According to Lee (13), the correlation of the variables and thus the Cramer’s V value was assessed as insignificant at a value of 0.0–0.1, as weak at a value of 0.1–0.2, as moderate at a value of 0.2–0.4, as relatively strong at a value of 0.4–0.6, as strong at a value of 0.6–0.8, and as very strong at a value of 0.8–1.0.

The entire project was reviewed and approved by the TiHo’s Data Protection Officer before final implementation. The participants of the survey, which was conducted via LimeSurvey, had to agree to the deposited data protection declaration in accordance with Art. 6 | 1 lit. e in conjunction with 89 GDPR, § 3 | 1 No. 1 NHG (Lower Saxony Higher Education Act) and § 13 NDSG (Lower Saxony Data Protection Act) before the questionnaire was carried out. The data collected from the questionnaire were evaluated and processed anonymously in accordance with the requirements of Art. 6 | 1 lit e, 89 GDPR in conjunction with § 13 NDSG.

## 3 Results

All participants are actively involved in teaching at the TiHo as academic staff and are summarized throughout the publication as lecturers or teaching staff for the sake of simplicity.

### 3.1 General personal details

In the survey, which was activated for four weeks in the period from 07.17.2020–08.14.2020, 174 lecturers of the scientific staff (*n* = 481) of the TiHo took part in the survey. The questionnaire was completed in full by 103 participants; only these results are included below. The response rate for the survey was 21.41%.

When asked about gender, 68 respondents (66.02%) stated that they were female and 32 respondents (31.07%) were male. A further 3 participants (2.91%) answered “No answer” to this question. The answer option “non-binary” was not selected by any of the teachers.

In terms of the age distribution of lecturers, the 30–39 age group was the largest with 42 staff members (40.78%), followed by the 50–59 age group, which included 22 lecturers (21.36%). Furthermore, at the time of the survey, 19 lecturers (18.45%) were in the 40–49 age group and 11 (10.68%) were in the under 30 age group. In addition, nine lecturers (8.74%) stated that they were aged 60 or over.

When asked about the assignment of the respective subject area, 42 participants (40.78%) indicated the subject area “Clinical training,” 32 participants (31.07%) the subject area “Paraclinical training,” 12 participants (11.65%) the subject area “Intermediary preclinical examination course” (Physikum), and six participants (5.83%) the subject area “Preliminary preclinical examination course” (Vorphysikum). A total of 11 respondents (10.86%) chose the answer “No answer.”

A total of 50 participants (48.54%) classified themselves as research associates, 22 participants (21.36%) stated that they held the position of working group leader, and 14 participants (13.59%) the position of institute/clinic director. Seven participants (6.80%) stated that they had the status of assistant doctor, five participants (4.85%) belonged to the group of private lecturers and one participant (0.97%) answered this question with the response “resident” and “other.” Three participants (2.91%) chose the option “No response.”

With regard to their active time in teaching, 47 lecturers (45.63%) stated that they had been actively involved in teaching for over 10 years. Furthermore, 35 lecturers (33.98%) chose the answer “1–5 years,” 18 lecturers (17.48%) chose the option “6–10 years“, and two lecturers (1.94%) chose the answer “< 1 year.” One teacher (0.97%) selected the answer “No response.”

A graphical representation of the general personal data is shown in Supplementary Figure S1.

### 3.2 Start of the digital semester

**Figure 1:**
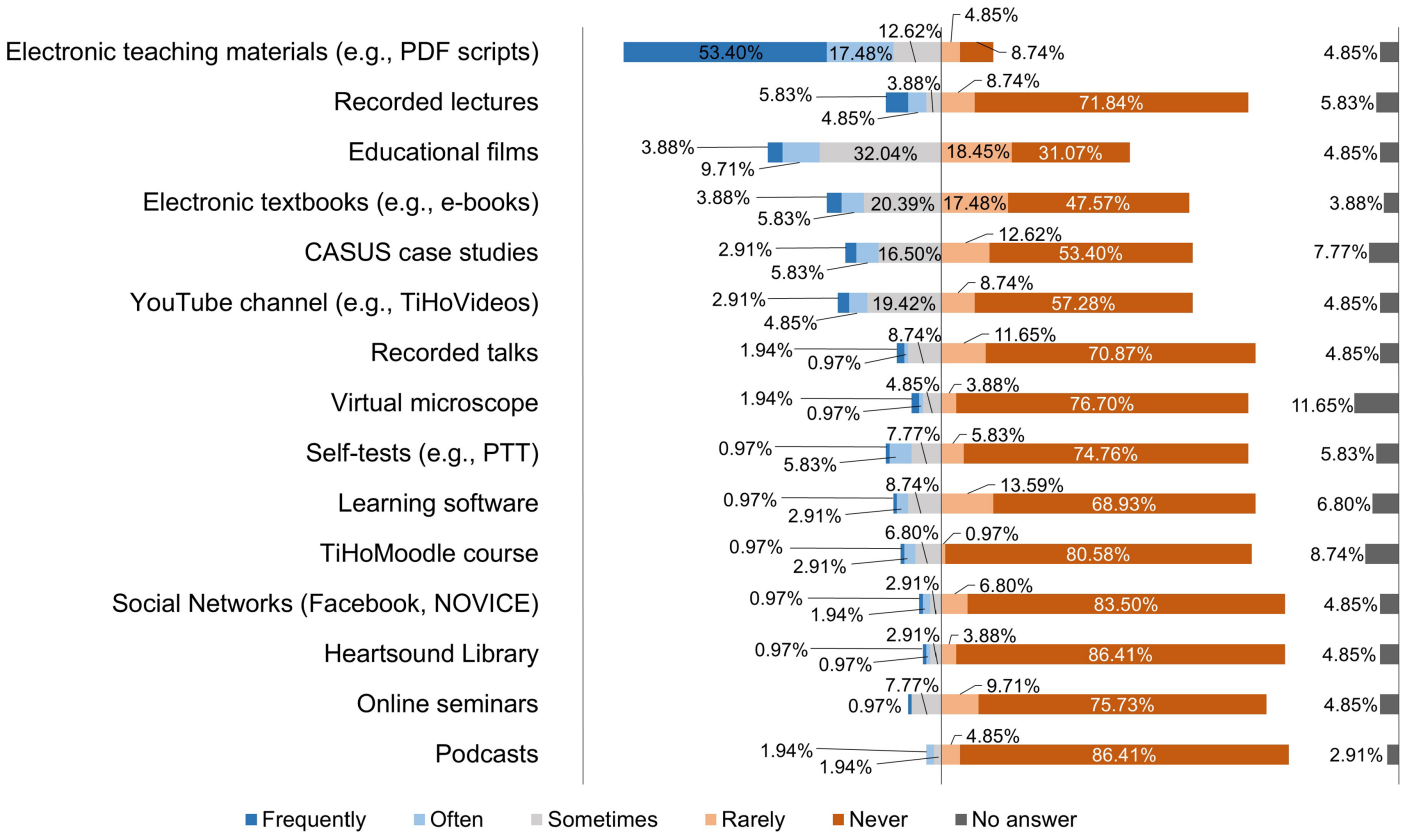
Online survey of teaching staff at the University of Veterinary Medicine Hannover, Foundation. Responses to the question: “How often did you use the following digital courses as part of your teaching activities at the TiHo BEFORE the switch to the digital semester?” (*n* = 103).

When asked how often teachers used digital courses in their teaching at the TiHo before the switch to the digital semester, it became clear that teachers at the TiHo had previously mainly used electronic teaching materials such as PDF scripts and educational films for their teaching. All results of this question are shown in Figure 1.

**Figure 2:**
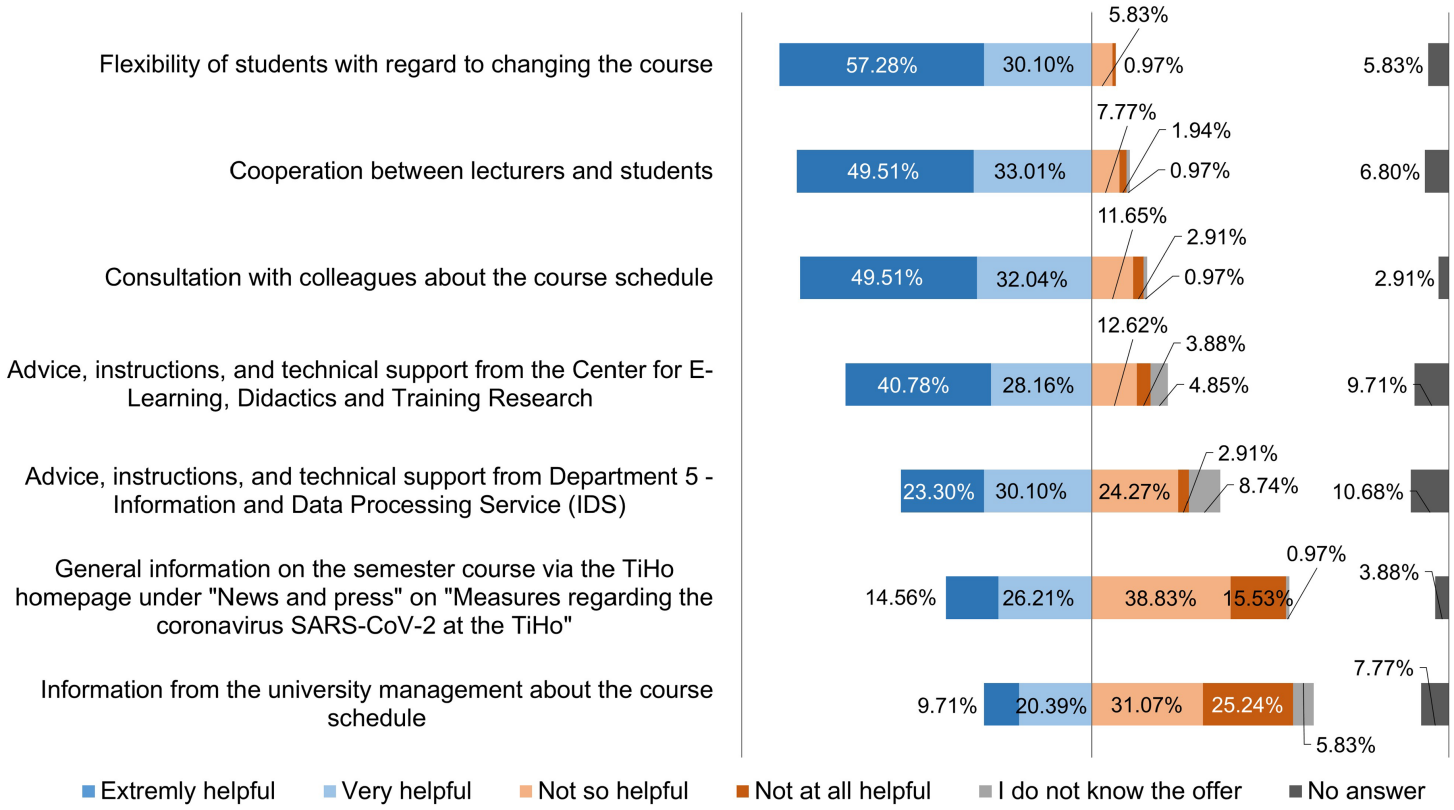
Online survey of teaching staff at the University of Veterinary Medicine Hannover, Foundation. Responses to the question: “What proved to be helpful in the initial phase of the semester? (*n* = 103).

**Figure 3:**
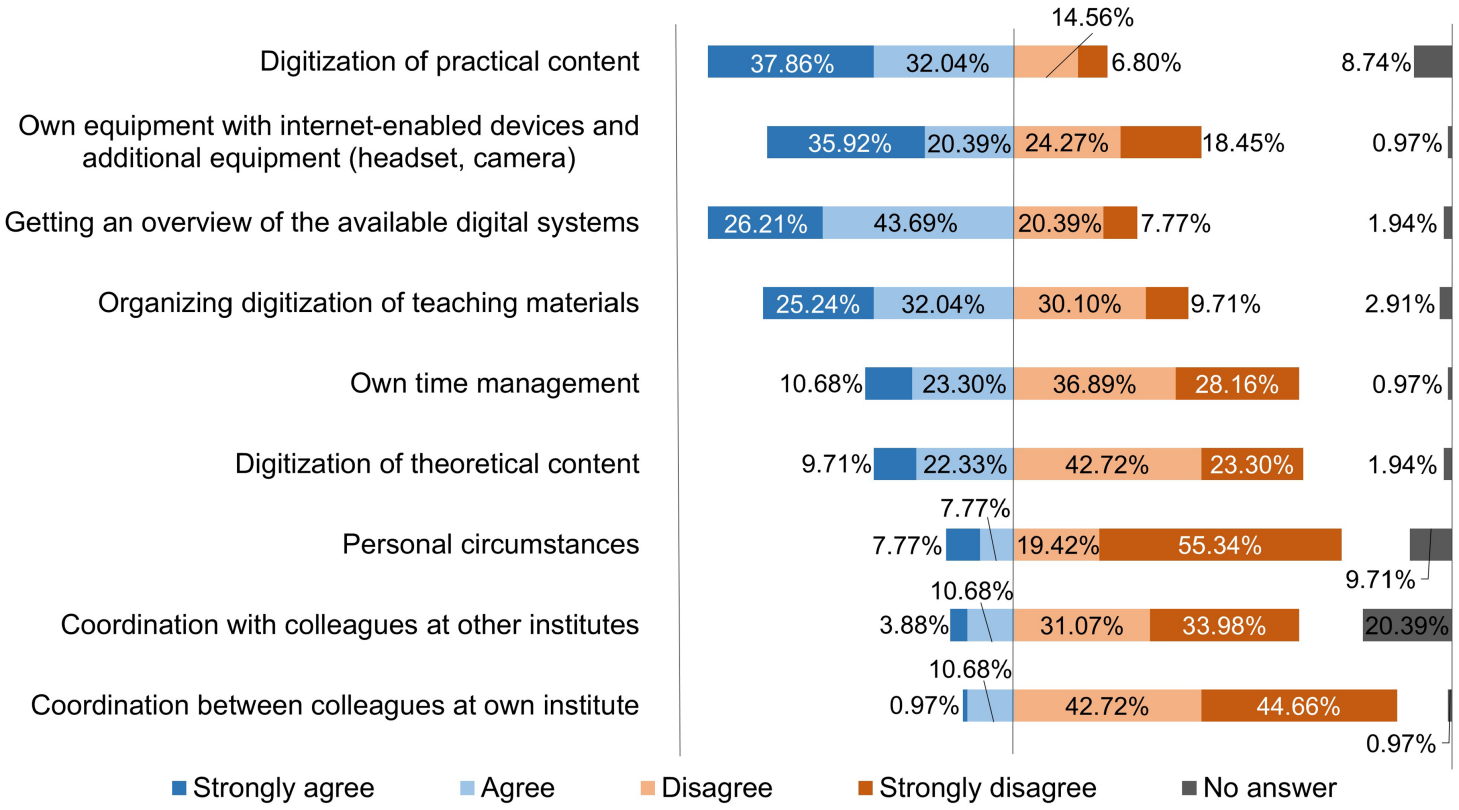
Online survey of teaching staff at the University of Veterinary Medicine Hannover, Foundation. Responses to the question: “What caused you difficulties in the initial phase of the semester? Please give your assessment of the following points.” (*n* = 103).

The teachers were asked what proved to be helpful in the initial phase of the semester and what caused them difficulties in the initial phase of the semester. The results thereof are shown in Figures 2, 3.

A statistical evaluation using a contingency table and Cramer’s V-test was used to check whether those involved in individual courses had major problems digitizing practical content. This statistical evaluation was also carried out for difficulties in digitizing theoretical content and then compared with each other (Tables 1, 2).

**Table 1:** Contingency table with frequencies of the linked characteristics subject area and problems with the digitization of practical content shown as absolute frequencies; row percentages in brackets (*n* = 86).

	Hardly/no difficulties in digitizing practical content (hardly/not at all applicable)	Difficulties with the digitization of practical content (true/mostly true)	Total
Clinical	6 (15%)	**34 (85%)**	40
Paraclinical	12 (38.71%)	**19 (61.29%)**	31
Intermediary preclinical examination course (Physikum)	1 (8.33%)	**11 (91.67%)**	12
Preliminary preclinical examination course (Vorphysikum)	0 (0%)	**3 (100%)**	3
Total	19	67	86

The values in bold are the highest percentage values in a row.

**Table 2:** Contingency table with frequencies of the linked characteristics subject area and problems with the digitization of theoretical content shown as absolute frequencies; row percentages in brackets (*n* = 91).

	Hardly/no difficulties in digitizing theoretical content (hardly/not at all applicable)	Difficulties in the digitization of theoretical content (true/mostly true)	Total
Clinical course	**28 (66.67%)**	14 (33.33%)	42
Paraclinical course	**24 (77.42%)**	7 (22.58%)	31
Intermediary preclinical examination course (Physikum)	**7 (58.33%)**	5 (41.67%)	12
Preliminary preclinical examination course (Vorphysikum)	**5 (83.33%)**	1 (16.67%)	6
Total	64	27	91

The values in bold are the highest percentage values in a row.

With a Cramer’s V value of 0.31, the correlation between the subject area and the difficulty in digitizing practical content (Table 1) can be classified as moderate according to Lee (13). A detailed analysis of the results presented in the contingency table (Table 1) shows that the percentage row frequencies in all subject areas for the response area “Difficulties in digitizing practical content (agree/ mostly agree)” were well above 50%. The results of the survey thus show that the respondents predominantly had difficulties with the digitization of practical content regardless of the subject area. With 19 lecturers (61.29%), those involved with teaching the “Paraclinical” course had the least difficulties in digitizing practical content.

With a Cramer’s V value of 0.16, the correlation between the subject area and the digitization of theoretical content (Table 2) could only be rated as weak according to Lee (13). A detailed analysis of the results presented in the contingency table (Table 2) shows that the majority of respondents in all subject areas stated that they had little to no difficulty in digitizing theoretical content. In comparison with the evaluation of the responses by subject area on the difficulties in digitizing practical content, however, the values here were clearly more evenly distributed, which was also confirmed by the weak Cramer’s V value.

### 3.3 Technical requirements

**Figure 4:**
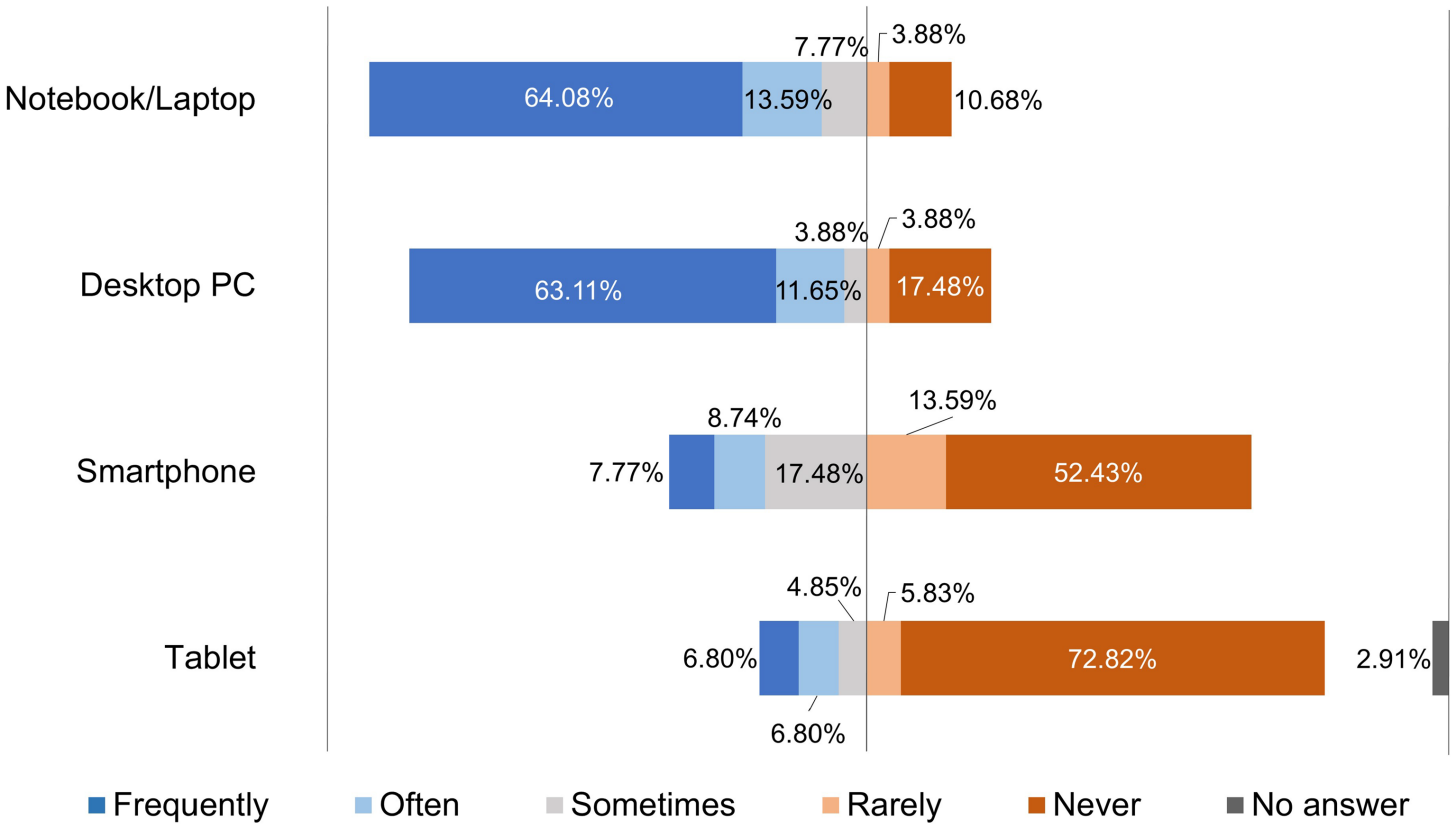
Online survey of teaching staff at the University of Veterinary Medicine Hannover, Foundation. Responses to the question: “Please indicate how often you use the following internet-enabled devices for teaching in the digital semester.” (*n* = 103).

Figure 4 shows how often the lecturers used the internet-enabled devices notebook/laptop, desktop PC, smartphone or tablet for their teaching in the digital semester. Lecturers at the TiHo used notebooks/laptops most frequently.

Teaching staff were also asked in the “Technical requirements” section what additional technical equipment they had available, with multiple choices being possible. The results showed that 81 participants (78.64%) had a headset, 72 (69.90%) had a printer, 66 (64.08%) had external storage media, 60 (58.25%) had a webcam, and 56 participants (54.37%) had scanners. In addition, 55 respondents (53.40%) stated that they were able to use speakers or integrated loudspeaker systems. A further 46 of the teaching staff (44.66%) stated that they had a microphone and 38 (36.89%) that they had headphones available. In addition to the options given, seven of the teaching staff (6.80%) chose the answer “Other.”

### 3.4 Teaching in the digital semester

**Figure 5:**
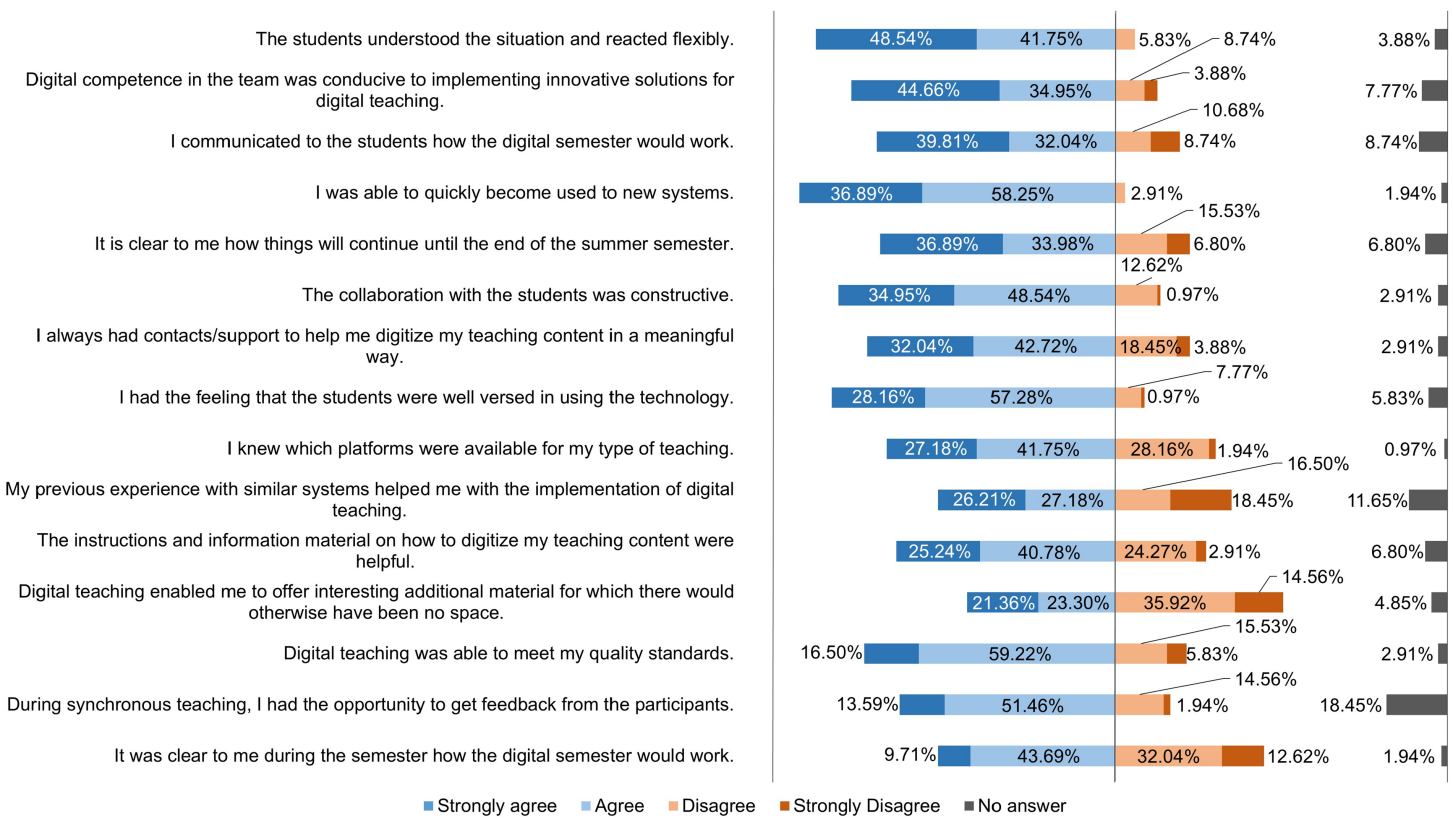
Online survey of teaching staff at the University of Veterinary Medicine Hannover, Foundation. Responses to the question: “Please rate the following statements.” (*n* = 103).

Figure 5 depicts how the lecturers rated various statements on teaching in the digital semester.

Concerning the question “Digital teaching currently takes place both synchronously in the form of live lectures and live question and answer sessions and asynchronously in the form of digital learning material, assignments, and learning control units. What is the best way for you to teach?” 63 respondents (61.17%), the majority of participants, chose the answer “Combination of synchronous and asynchronous teaching.” A total of 25 teaching staff members (24.27%) stated that they could teach best with “Synchronous teaching,” 10 participants (9.71%) chose the answer option “Asynchronous teaching”, and a further five respondents (5.85%) stated “I do not know.”

Using a contingency table and the Cramer’s V-value, the results of the survey of teaching staff members at the TiHo were used to test whether there was a correlation between the age or teaching experience of the lecturers and the preferred teaching system and how strong this correlation was.

The two contingency tables, Tables 3, 4, show the relationship between the age of the teaching staff and the preferred format of digital teaching, or the teaching experience and the preferred form of digital teaching. A Cramer’s V of 0.26 was determined in the investigation of the preferred form of digital teaching compared with the teaching age, and a Cramer’s V of 0.23 was determined in the investigation of teaching experience compared with the preferred form of digital teaching. According to Lee (13), the two determined Cramer’s V values each showed a moderate correlation between the variables investigated. Descriptively, Table 3 shows that in the age groups <30 years, 30–39 years, 40–49 years, and 50–59 years, the combination of synchronous and asynchronous teaching was named by a high percentage of respondents as the form of teaching with which they were best able to implement their teaching. In the 60+ age group, on the other hand, synchronous teaching was stated as the preferred form of teaching by five lecturers (55.56%) who formed the majority in this group. A descriptive analysis in Table 4 shows that teachers with teaching experience of 1–5 years, 6–10 years, and > 10 years also preferred the combination of synchronous and asynchronous teaching. Only in the group of teaching staff with teaching experience of <1 year, in which only two respondents were represented, did a descriptive evaluation of the contingency table show a different percentage majority. In this group, one of two respondents chose the answer “synchronous teaching” and one of two respondents chose the answer “asynchronous teaching.

**Table 3:** Contingency table with frequencies of the linked characteristics of lecturers’ age and preferred digital teaching format shown as absolute frequencies; row percentages in brackets (*n* = 98).

	Synchronous teaching	Asynchronous teaching	Combination of synchronous and asynchronous teaching	Total
< 30 years	2 (22.22%)	3 (33.33%)	**4 (44.44%)**	9
30–39 years	10 (25.64%)	3 (7.69%)	**26 (66.67%)**	39
40–49 years	2 (10.53%)	2 (10.53%)	**15 (78.95%)**	19
50–59 years	6 (27.27%)	1 (4.55%)	**15 (68.18%)**	22
60 + years	**5 (55.56%)**	1 (1.11%)	3 (3.33%)	9
Total	25	10	63	98

The values in bold are the highest percentage values in a row.

**Table 4:** Contingency table with frequencies of the linked characteristics of lecturers’ teaching experience in years and preferred digital teaching format shown as absolute frequencies; row percentages in brackets (*n* = 97).

	Synchronous teaching	Asynchronous teaching	Combination of synchronous and asynchronous teaching	Total
< 1 year	**1 (50%)**	**1 (50%)**	0 (0%)	2
1–5 years	5 (15.63%)	4 (12.50%)	**23 (71.88%)**	32
6–10 years	4 (25%)	3 (18.75%)	**9 (56.25%)**	16
> 10 years	15 (31.91%)	2 (4.26%)	**30 (63.83%)**	47
Total	25	10	62	97

The values in bold are the highest percentage values in a row.

**Figure 6:**
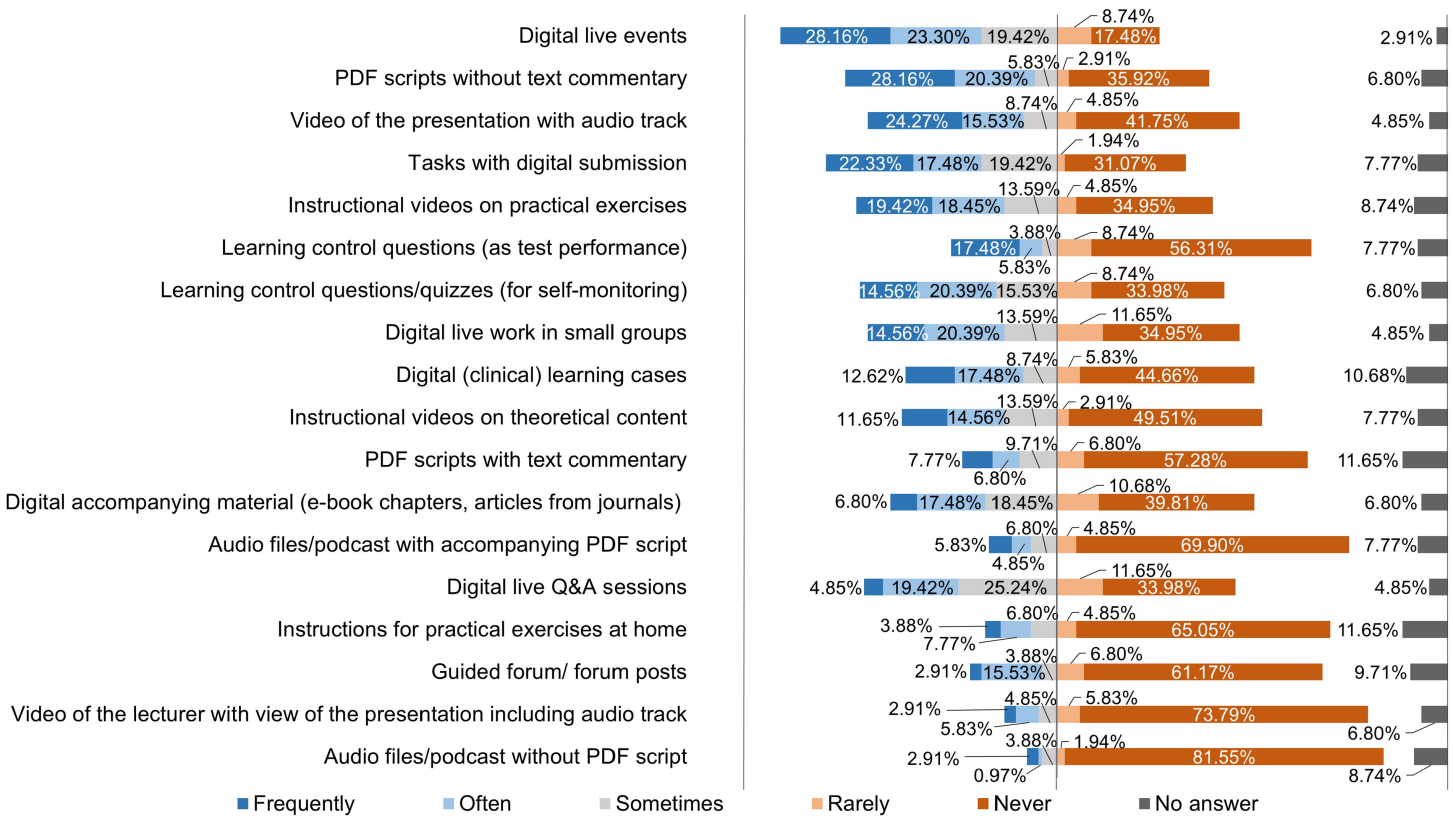
Online survey of teaching staff at the University of Veterinary Medicine Hannover, Foundation. Responses to the question: “How often did you use the following digital teaching services as part of your teaching activities at the TiHo DURING the digital semester?” (*n* = 103).

Figure 6 shows how often the lecturers used different course offerings as part of their teaching at the TiHo during the digital semester.

**Figure 7:**
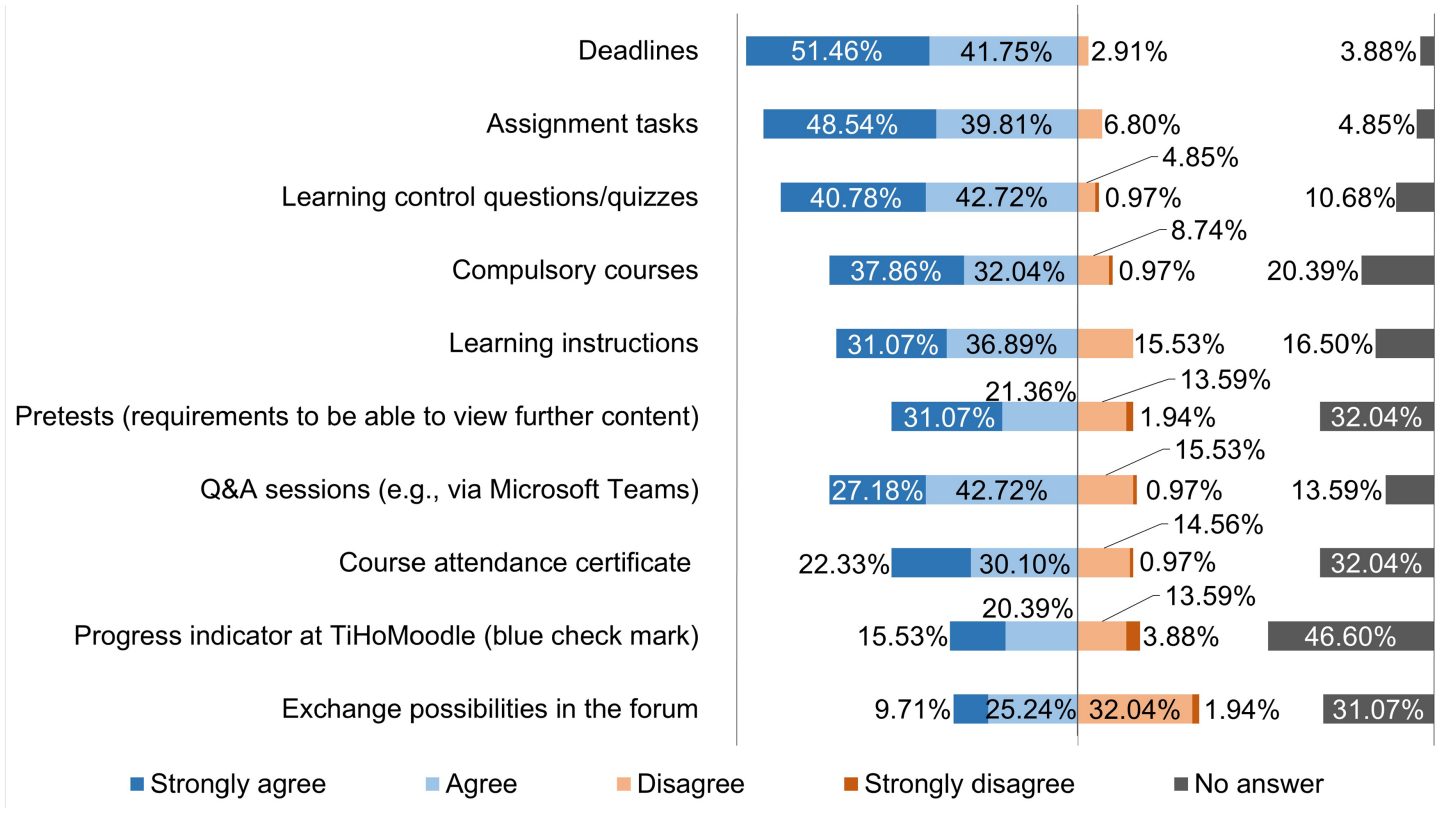
Online survey of teaching staff at the University of Veterinary Medicine Hannover, Foundation. Responses to the question: “In your opinion, what measures help students to process digital material promptly?” (*n* = 103).

Figure 7 shows which measures teaching staff believe are helpful for students to process digital material promptly.

When asked how the lectures should be recorded, 18 lecturers (17.48%) opted for a recording as a whole and 31 lecturers (30.10%) for “no answer.” With 54 respondents (52.43%), the majority of lecturers opted to record the lectures in chapters.

The 54 lecturers (52.43%) who had opted for the answer “In chapters” in the previous question were asked in a follow-up question how long the individual chapters should be. A total of 29 respondents (53.70%) opted for a duration of 11–20 min, 11 (20.37%) for 21–30 min, nine (16.67%) for 0–10 min, and a further three lecturers (5.56%) for 31–40 min. One person each (1.85%) chose the answer options “41–50 min” and “51–60 min.”

### 3.5 Effects of the digital semester on teaching

When asked whether the changeover to a digital semester generally had a negative impact on lecturers, 53 participants (51.46%) answered “No,” while 44 lecturers (42.72%) answered in the affirmative. A total of six respondents (5.83%) chose “No answer.”

A total of 78 lecturers (75.73%) stated that the effort required for teaching digitally was higher compared to conventional face-to-face events with teaching materials (e.g., scripts). For 22 participants (21.36%) the effort was the same and for two respondents (1.94%) the effort was lower. One respondent (0.97%) selected “no answer.”

**Figure 8:**
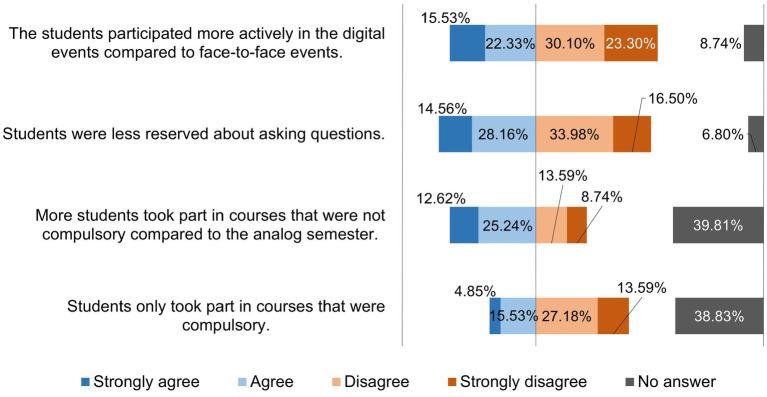
Online survey of teaching staff at the University of Veterinary Medicine Hannover, Foundation. Responses to the question: “Please evaluate the following statements on the learning behavior and active participation of students in the digital semester.” (*n* = 103).

**Figure 9:**
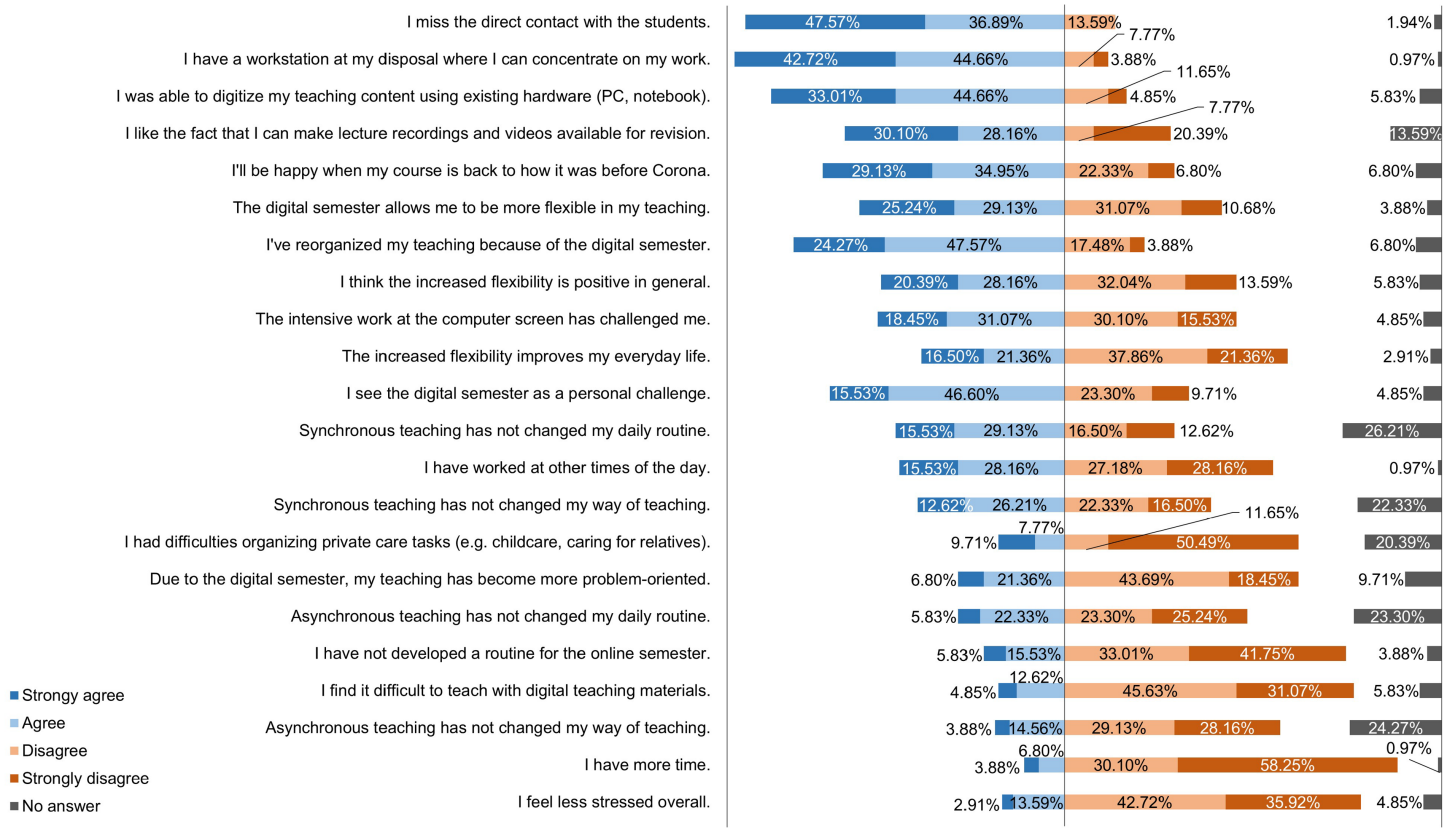
Online survey of teaching staff at the University of Veterinary Medicine Hannover, Foundation. Responses to the question: “Please rate the following statements about your flexibility | self-organization | resilience in the digital semester.” (*n* = 103).

Figure 8 shows how the lecturers rated various statements on learning behavior and the active participation of students in the digital semester. Figure 9 shows the lecturers’ responses to statements about their flexibility, self-organization, and resilience in the digital semester.

The question “Have you encountered any other challenges this semester?” was answered in the negative by 55 lecturers (53.40%), while 30 respondents (29.13%) chose the answer option “Yes, namely:” and entered other challenges as a free text response. A total of 18 participants (17.48%) chose the answer “no answer.” A total of 10 response categories were formed when categorizing the free text responses. With seven responses each, the categories “Lack of additional equipment” and “High workload” were mentioned most frequently as challenges. The organizational effort category was mentioned a total of five times. COVID-19 crisis management, restrictions in own teaching, childcare, and working at home were cited as challenges with three mentions each. Two mentions could be assigned to the category of course planning. In addition, two further categories were formed, each with one individual mention.

### 3.6 Outlook and general suggestions

**Figure 10:**
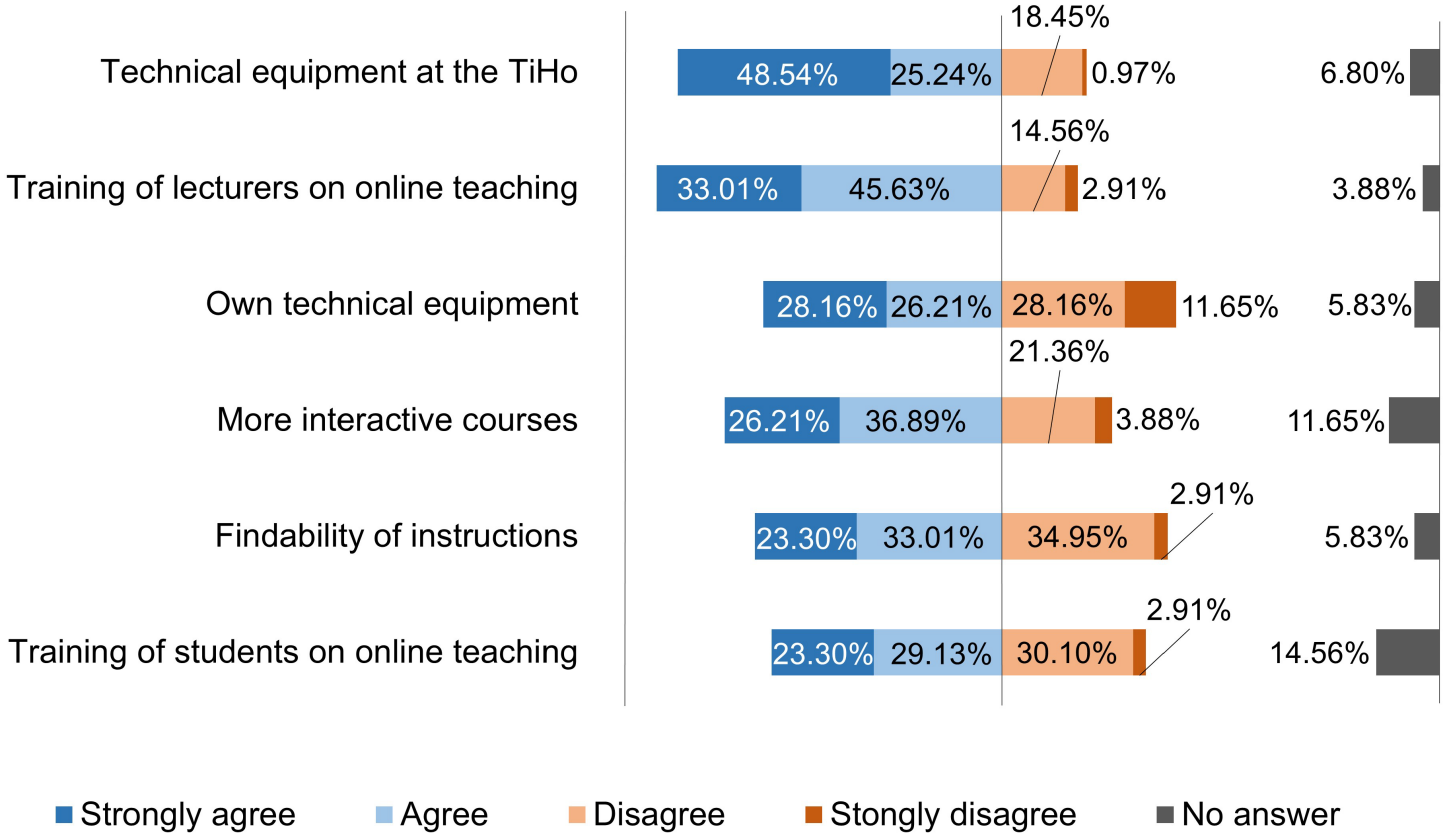
Online survey of teaching staff at the University of Veterinary Medicine Hannover, Foundation. Responses to the question: “Where do you see room for improvement with regard to digital teaching?” (*n* = 103).

Figure 10 shows where teachers see a need for improvement with regard to digital teaching.

In response to the question “Would you welcome the sustainability of the following systems (after COVID-19 pandemic)?,” 60 teachers (58.25%) answered “strongly agree,” 27 participants (26.21%) “agree,” 11 respondents (10.68%) “disagree“, and one person (0.97%) “strongly disagree” with regard to the Microsoft® Teams platform. A further four participants (3.88%) opted for the “no answer” option. With regard to the TiHoMoodle learning management system, 43 lecturers (41.75%) answered “strongly agree” with regard to the sustainability of the system after the COVID-19 pandemic. A total of 17 participants (16.50%) opted for “agree,” eight respondents (7.77%) for “disagree”, and four people (3.88%) for “strongly disagree.” A total of 31 teachers (30.10%) abstained from this sub-question and chose the answer “No answer.”

**Figure 11:**
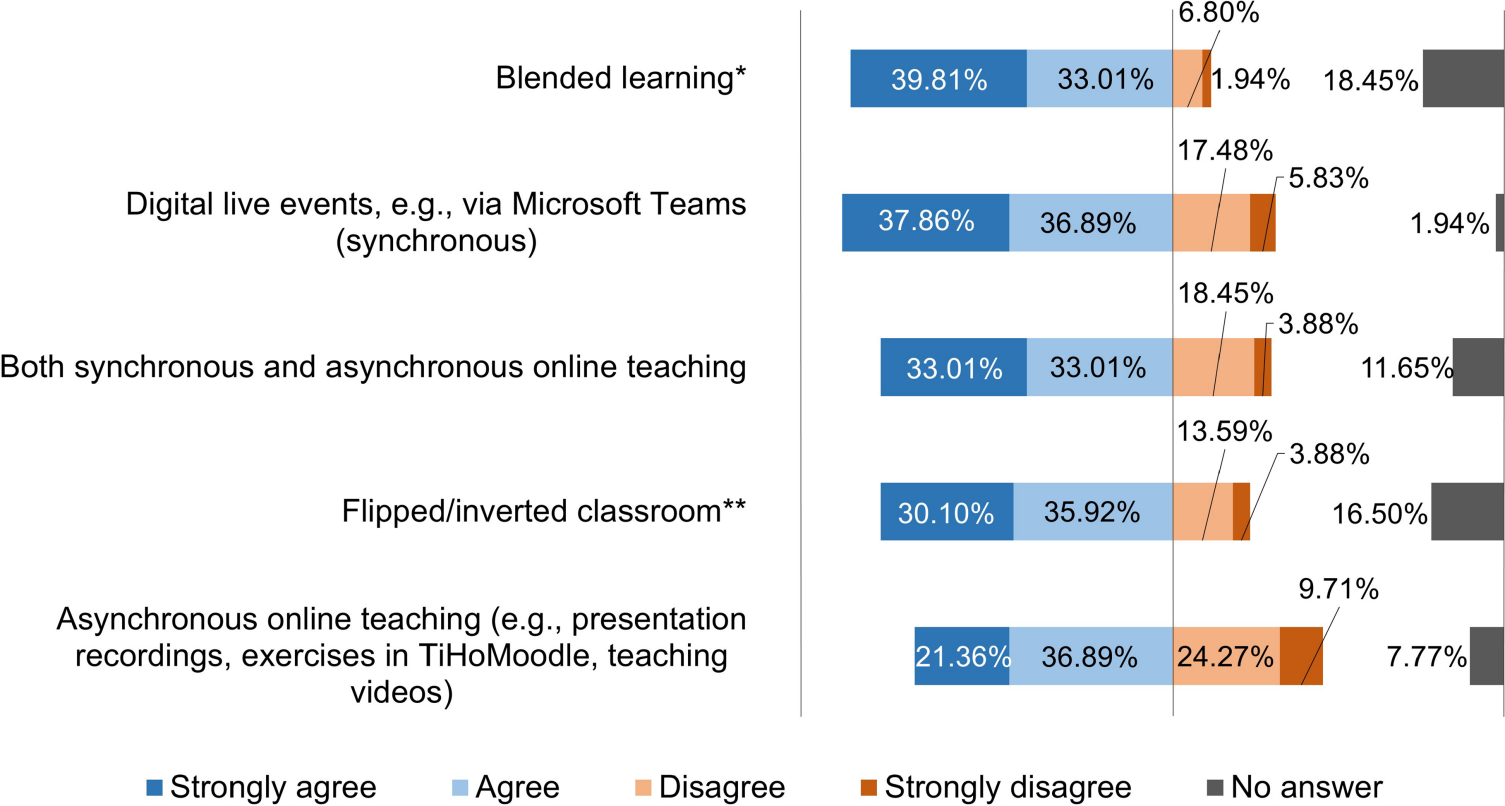
Online survey of teaching staff at the University of Veterinary Medicine Hannover, Foundation. Responses to the question: “Which teaching method do you see the greatest potential in the future?” (*n* = 103); *Combination of classroom teaching and online units; **Combination of initial preparation of learning content (e.g., watching lecture recordings), and subsequent discussion of questions.

The teaching staff were asked to rate five different teaching methods on a four-point rating scale by indicating which method they see the greatest potential for the future. The results of this survey are shown in Figure 11.

In addition, three free text questions were formulated at the end of the questionnaire, which could be answered optionally and were evaluated by means of a categorization of the answers. A total of 37 teachers (35.92%) responded to the first of these three questions “What did you particularly like in this digital semester?” A total of nine categories were identified when evaluating the responses. With 15 responses, the category of cohesion/willingness/support/motivation was mentioned most frequently. With 14 mentions, lecturers also liked the use of new systems in the digital semester. The category of increased flexibility also received 14 mentions. Of these 14 responses, seven referred to the flexibility and repetition provided by the digital teaching material for students. A total of 11 responses each could be assigned to the category implementation of digital teaching. Lecturers also liked the positive feedback from students, which was mentioned in 10 statements. A total of eight responses could be assigned to the category of positive effects of digital teaching. Two of these referred to system reflection and breaking old patterns of behavior. Six responses could be assigned to technical support from the information and data processing service and e-learning advice. Two responses each fell into the categories of communication. A further category was formed from a single response.

A total of 32 lecturers (31.07%) responded to the optional question “Do you have any suggestions for improving digital teaching?” The evaluation of the statements resulted in a division into five categories. With 14 responses, technical equipment was the most frequently mentioned suggestion for improvement. In particular, the technical equipment of employees stood out with a total of seven mentions in this category. A total of 11 mentions could be assigned to the category of promoting and expanding digital teaching systems. Of these, lecturers requested a sustainable training program for lecturers in four statements and better instructions in three statements. The suggestion for improvement of better communication and organization of teaching was also mentioned 11 times. Of these, the lecturers explicitly mentioned better planning of the semester in four statements. Four mentions fell into the category of strengthening participation processes. In addition, there was one further category with a single mention.

Finally, the responses of the 33 lecturers (32.04%) to the question “What technology/software did you miss for teaching digital courses in the summer semester 2020?” were divided into six different categories. With a total of 26 mentions, the category of technical equipment was mentioned, which the lecturers missed for the implementation of their digital courses. Of these, two mentions related to technical equipment when working from home. Eight statements fell into the category of other tools, which included apps, software, learning boxes as well as other video conference systems and online offerings. In a total of six responses, teaching staff missed the stability of existing systems. A further two responses could be assigned to the category of earlier introduction of new systems. The evaluation of the responses also revealed a further category with one mention.

## 4 Discussion

The fully completed questionnaires already provide a good insight into the experiences of lecturers at the TiHo with regard to the changeover to an exclusively digital summer semester 2020, although only 103 (59.10%) of the 174 completed questionnaires were fully completed. The response rate for fully completed questionnaires was therefore 21.41%. However, it should be noted that the e-mail distribution list also included people who were not actively involved in teaching and were therefore not addressed by the survey. As some studies have shown, long questionnaires often lead to an increased dropout rate (14, 15). The high dropout rate of 71 questionnaires (40.80%) can be attributed, among other things, to the relatively long completion time of approx. 40 min, especially in a time that was already challenging due to the COVID-19 pandemic. Our research revealed that there are only a limited number of publications currently available in the field of veterinary medicine and medical education that examine the perspectives of teaching staff at universities during the pandemic, so this study fills a gap in the field of veterinary medicine.

The rapid transformation of media didactics due to the *ad hoc* switch to a purely digital summer semester 2020 not only posed new challenges for the information technology and e-learning facilities of the universities and demanded increased support, but also revealed itself in the difficulty of suddenly procuring software, hardware, and licenses to a sufficient extent (8). In this context, the survey of TiHo lecturers showed that a good technical infrastructure had already been established before the pandemic and that, as a result of funding from third-party projects such as KELDAT (16), eCULT+ (17), and FERTHIK (18), there was a solid knowledge of e-learning and a willingness to use digital learning technology in teaching. Digital teaching materials were also existing and in use at many other veterinary educational institutions before the pandemic (19). A clear majority of lecturers at the TiHo stated in the survey that they were able to digitize their teaching content with the existing hardware and software, which was confirmed by the teaching evaluation of the following winter semester 2020/2021 and summer semester 2021 (20).

The area of technical equipment was rated by many lecturers (73.78%) as being in need of improvement. The surveys of teaching staff, support staff, and university management in the studies by Bosse et al. and Hense and Goertz (21, 22) showed a similar picture of the technical infrastructure. From a didactic point of view, there is a particular need for optimization in the area of additional technical equipment, such as a webcam, which only about half (58.25%) of the lecturers at the TiHo had in order to establish successful social communication between lecturers and students in synchronous but also asynchronous online teaching, according to Kerres (23). With regard to equipping lecturers with webcams and headsets, however, an improvement was already noted at the TiHo in the follow-up survey (20). However, it should be noted that the number of completed questionnaires was significantly lower in the follow-up survey (87) and the comparison therefore only shows a trend.

In addition to possibilities for successful social communication in digital teaching, the study by Kerres, 2022 also reveals many other strategies for how teachers can optimize both synchronous and asynchronous digital teaching, as the predominantly face-to-face teaching that has been used to date cannot be converted 1:1 to digital teaching (23). Motivating and stimulating students as well as selecting and using the right digital tools are particularly important in asynchronous teaching. The fact that there is a need for further training in this field was also expressed by 78.64% of participants in this survey, with a desire for training opportunities for online teaching, as well as by 70.11% of respondents in the following study by Naundorf (23). The studies by Bosse et al. and Hense and Goertz also showed an increased need among teachers for further development measures, such as information materials, instructions, and workshops at the beginning of the COVID-19 pandemic. Another study in the field of medical education also showed that teaching staff stated that they had deficits in the comprehensive use of digital teaching methods during the COVID-19 pandemic (24). These deficits could be reduced through further development measures and training opportunities, thus further optimizing digital teaching.

Students at the TiHo also expressed the wish that teaching staff should receive more training in the use of digital learning technologies (25). The teaching of digital skills is therefore being promoted at the TiHo and integrated into the didactic qualification measures for teaching staff as set out in the document ESEVT SOP 2023 (11).

In contrast to the experiences of lecturers at other universities, 65.05% of lecturers at the TiHo stated that they had a very good or good opportunity to receive feedback from students in the synchronous courses, and in 10 free text responses, the feedback was also rated as positive overall. Despite this predominantly positive assessment of the lecturers with regard to the feedback, the lecturers (37.86%) evaluated the participation of the students in the digital courses as no more active than previously in the face-to-face courses. The follow-up survey at the TiHo in the subsequent hybrid semesters showed an almost identical picture of the evaluation of active participation from students in synchronous courses compared to face-to-face courses (36.78%) (20). Other studies revealed a rather controversial experience in this regard among lecturers who cited the lack of interactivity and the difficulty of implementing the dynamics that develop in lectures in digital teaching as a challenge (8). However, even though the feedback from the students was evaluated as positive by the lecturers, the results of the survey clearly showed that the lecturers at the TiHo still missed direct contact with the students. This was not only consistent with the results from the follow-up survey (20), but also with the experiences of lecturers in other studies (7, 8, 26).

The crisis situation of the pandemic created high pressure to act and the expectation to find pragmatic solutions quickly, so that many lecturers at the beginning of the semester mainly used teaching methods they were already used to (7). As can be seen in Figure 6, lecturers at the TiHo preferred to use synchronous live events via Microsoft® Teams (51.46%) and upload PDF documents to platforms (48.55%), followed by lecture recordings (39.80%), and 15 other formats. The conditions at the beginning of summer semester 2020 were far from ideal due to the *ad hoc* changeover and cannot be compared with the regular conception and planning processes for implementing digital teaching formats (27). Didactic planning for digital teaching was requested by all heads of institutes at the TiHo at the beginning of the pandemic. This planning was reviewed and adapted as necessary, which contributed to the fact that lecturers proved to be increasingly willing to try out new digital tools and teaching formats as the semester progressed. This can be seen especially clearly in a comparison of the use of teaching formats before the pandemic (Figure 1) and during the digital summer semester 2020 (Figure 4). While 10.68% of lecturers used lecture recordings very frequently or frequently for their teaching before the pandemic, this figure increased to 39.8% during the digital semester. Educational videos were only used very frequently or frequently for teaching by 13.59% of lecturers before COVID-19 pandemic, while in the digital semester, 37.87% of lecturers used educational videos for practical exercises and 26.30% used them very frequently or frequently for theoretical content. A similar picture can be seen in the use of interactive learning cases and additional digital material (e.g., e-books). In addition, newly introduced digital offerings such as synchronous live events (51.46%) and podcasts (10.68%) were also used very frequently to frequently by lecturers for their teaching. A total of 71.84% of lecturers at the TiHo even stated that they had largely reorganized their teaching.

In contrast, the survey of the following two hybrid semesters showed that lecturers at the TiHo still preferred to use synchronous live events (67.82%) for their teaching, but that the use of asynchronous formats [e.g., instructional videos for practical content (34.49%) and instructional videos on theoretical content (17.24%)], with the exception of assignments with digital delivery (45.98%), was used considerably less (20). These results are in contrast to the preferred teaching system of a combination of synchronous and asynchronous teaching, which was clearly preferred by teachers at the TiHo in both surveys and should be an indication for further training opportunities, as the use of asynchronous formats in teaching has certainly proven to be successful, as confirmed by the study by Gomez et al., among others (28). In Naundorf’s study (23), the majority of lecturers (64.37%) also stated that they had completely reorganized their teaching. This shows that teaching at the TiHo is also still in a discovery phase with regard to the most suitable digital teaching methods for the various subjects.

In the search for the best digital teaching formats for the respective forms of teaching, it became clear that practical content in particular, such as laboratory courses, dissection courses, exercises on animals, and practicals, were difficult to implement in purely digital form, even if these were possible in small groups and under strict hygiene requirements at the TiHo toward the end of the summer semester 2020. This was consistently evident in all subject areas (Tables 1, 2) and is also largely consistent with the results of the follow-up study, although the teaching of practical content during the pandemic was rated as slightly less difficult (20), as even more practical exercises were possible again. A further study also showed that the teaching of clinical skills in presence was resumed as quickly as possible at many other veterinary training centers under the appropriate hygiene and protection regulations (29). So not only the experience of teaching staff at the TiHo, but also other studies have shown the indispensability of university classroom teaching in practical training (7, 8, 24). There should be a change in thinking and a new appreciation of teaching time for the practical training of students in the future. Blended learning as a combination of various digital and analog teaching formats as well as the flipped classroom as a special implementation of blended learning can support the preparation and follow-up of practical courses to make optimal and efficient use of the time in attendance for learning practical skills (30–32). However, it is not only important to know the theory of these methods, but also to be able to implement them. For example, an international study in which veterinary teaching staff from the USA, UK, and Australia were surveyed showed that 95% of teaching staff were aware of the flipped classroom method, while 64% of them actively implemented this method in their teaching (33). It is important to recognize that the creation and implementation of sustainable didactic concepts, especially for digital asynchronous teaching, requires time for planning and further training, which should be allocated to teaching staff.

This increased time requirement for digital teaching was also reflected in the responses from lecturers at the TiHo. The results of the survey (75.73%), like other studies, showed that the effort required for digital teaching is considerably higher compared to the usual face-to-face courses (7, 8). On the positive side, it should be mentioned that in the follow-up survey at the TiHo, significantly fewer lecturers perceived the effort required for digital teaching as higher (44.83%) and significantly more lecturers even perceived the effort as lower (12.64%) (20), which can be explained by the essential advantage of the reusability of digital material. However, even though lecturers perceived the effort required for digital teaching to be lower in the two subsequent semesters (23), the initially increased workload for lecturers can be explained primarily by the *ad hoc* switching to purely digital teaching at the beginning of the summer semester 2020. In addition, the COVID-19 pandemic also brought further challenges, such as parallel work in the clinic or teaching, which were also restricted and made more difficult by the pandemic (34).

The participants rated blended learning as the teaching method with the greatest potential for the future. This conclusion is also clear from the 61.17% of teaching staff at the TiHo who stated that they were best able to teach with a combination of synchronous and asynchronous teaching. The result differed in the follow-up survey at the TiHo. It showed that although slightly more participants rated blended learning as the teaching method with the greatest potential for future teaching, only 50.57% of lecturers still stated that they were best able to teach with a mixture of synchronous and asynchronous teaching compared to the survey on the two subsequent hybrid semesters at the TiHo (20). Furthermore, other studies came to the conclusion that university teaching staff see the greatest potential for the future in blended learning (7, 21, 22). Blended learning has many advantages compared to pure face-to-face teaching, not only due to its flexibility in terms of time and space, but also because it enables good interaction between lecturers and learners in asynchronous scenarios and it can reach a large number of students without requiring more teaching resources (35). Furthermore, it has been shown to increase student engagement, personal responsibility, and satisfaction (28, 36). In addition, digital resources are persistent and therefore easy to use over longer periods of time (30). Some studies have also shown that blended learning with a mixture of digital and face-to-face teaching not only has a positive effect on motivation (28, 37), but also on learning success (38) and students’ academic results (28, 39).

When using Cramer’s V to test whether there was a correlation between the age or teaching experience of the teachers and the preferred teaching system and how strong this correlation can be classified as, the two Cramer’s V values determined, according to Lee (13), showed a moderate correlation between the variables examined. While all age groups from <30 to 50–59 as well as teachers with teaching experience of 1–5 years, 6–10 years, and > 10 years named the combination of synchronous and asynchronous teaching as their preferred form of teaching, the majority of the 60+ age group named synchronous teaching. However, it should be noted in both tables (Tables 3, 4) that both the groups of teachers by age and by teaching experience were very small and the descriptive evaluations of the contingency tables should not be generalized.

Although the study at the TiHo clearly shows that lecturers see the enforced digital semester as an opportunity to further develop digital teaching, 64.08% of lecturers also stated that they would be happy if studies were to take place again in the same format as before the COVID-19 pandemic. This discrepancy is also evident in other surveys and can be explained, among other things, by the increased effort required to create digital teaching and the restrictions in communication, but also by the fact that digital teaching at universities is still in the consolidation phase and the extent to which digital teaching strategies can be established in the long term still needs to be determined (8, 26, 34, 40).

The results of the study show that the TiHo already had a good technical infrastructure at the beginning of the COVID-19 pandemic, but that there is an increased need for additional technical equipment such as webcams and headsets. In the 2020 summer semester, TiHo teaching staff mainly used digital formats that they were already used to, such as synchronous live events, PDF documents and lecture recordings. TiHo teaching staff saw a major difficulty in the digital implementation of practical veterinary training for students. The study also displayed that the creation of digital teaching requires more time compared to face-to-face teaching and that teachers have an increased need for training and further education on digital teaching. However, the teaching staff at the TiHo were able to digitize their teaching well with the resources available to them and see great potential for future veterinary training, especially in the “blended learning” teaching format. Furthermore, the results of the study are limited by the high dropout rate of 40.80%.

In summary, and with a view to veterinary education, there is a need for well thought-out didactic concepts and good practice examples that should be provided to teaching staff in order to accelerate the digital transformation process at veterinary educational institutions and to prevent a 100% reverting to traditional teaching formats. At the same time, as this study also shows, there is also a need for support in the form of training courses for teaching staff to strengthen their digital skills.

## Data availability statement

Data that are not protected by the GDPR are anonymized and accessible via the Supplementary Material.

## Author contributions

MK: Formal analysis, Methodology, Investigation, Conceptualization, Writing – original draft. AT: Supervision, Writing – review & editing. ES: Supervision, Methodology, Conceptualization, Writing – review & editing.

## Supplementary material

SUPPLEMENTARY DATA SHEET 1 Excerpt from the online questionnaire about the digital summer semester 020 for lecturers and teaching staff at the University of Veterinary Medicine Hannover, Foundation with the questions evaluated in the article.

SUPPLEMENTARY FIGURE 1 Online survey of teaching staff at the University of Veterinary Medicine Hannover, Foundation. Graphical representation of the general personal data (*n* = 103).

## References

[1] Hochschulrektorenkonferenz. (2020). Auflagen und Regelungen der Bundesländer für den Lehr- und Prüfungsbetrieb an Hochschulen im Sommersemester 2020. Available at: https://www.hrk.de/themen/hochschulsystem/covid-19-pandemie-und-die-hochschulen (Accessed April 27, 2023)

[2] Börchers M Tipold A Pfarrer C Fischer M Ehlers JP. Acceptance of case-based, interactive e-learning in veterinary medicine on the example of the CASUS system. Tierarztl Prax Ausg K Klientiere Heimtiere. (2010) 38:379–88. 22212751

[3] Kleinsorgen C Kankofer M Gradzki Z Mándoki M Bartha T Köckritz-Blickwede M. Utilization and acceptance of virtual patients in veterinary basic sciences – the vetVIP-project. GMS journal. Med Educ. (2017) 34:34. doi: 10.3205/zma001096, PMID: 28584867 PMC5450435

[4] Müller LR Tipold A Ehlers JP Schaper E. Digitalisierung der Lehre? – Begleitende Bedarfsanalyse zur Implementierung von Vorlesungsaufzeichnungen in der tiermedizinischen Ausbildung. Tierarztl Prax Ausg K Klientiere Heimtiere. (2019) 47:164–74. doi: 10.1055/a-0885-0834 31212349

[5] Müller LR Tipold A Ehlers JP Schaper E. TiHoVideos: veterinary students' utilization of instructional videos on clinical skills. BMC Vet Res. (2019) 15:326. doi: 10.1186/s12917-019-2079-2, PMID: 31506098 PMC6737648

[6] Nessler J Schaper E Tipold A. Proof of concept: game-based Mobile learning-the first experience with the app Actionbound as case-based geocaching in education of veterinary neurology. Front Vet Sci. (2021) 8:753903. doi: 10.3389/fvets.2021.753903, PMID: 34993245 PMC8724428

[7] Speidel R Schneider A Korner J Grab-Kroll C Ochsner W. Did video kill the XR star? Digital trends in medical education before and after the COVID-19 outbreak from the perspective of students and lecturers from the faculty of medicine at the University of Ulm. GMS J Med Educ. (2021) 38:1497. doi: 10.3205/zma001497, PMID: 34651059 PMC8493844

[8] Malewski S Engelmann S Erleben PL. Herausforderungen und zukünftige Lehrszenarien in der Online-Lehre: Eine Mixed-Method-Studie zum Covid-19 Sommersemester 2020 aus Sicht von Lehrenden. MedienPädagogik. (2021) 40:97–117. doi: 10.21240/mpaed/40/2021.11.12.X

[9] TAppV. (2016). Verordnung zur Approbation von Tierärztinnen und Tierärzten vom 27. 2006 (BGBl. I S. 1827), mit Änderungen durch Artikel 1 der Verordnung vom 20. Dezember 2016 (BGBl. Nr. 66, S. 3341). Available at: https://www.gesetze-im-internet.de/tappv/BJNR182700006.html (Accessed January 1, 2024)

[10] Directive. (2005). 2005/36/EC of the european parliament and of the council. Available at: http://data.europa.eu/eli/dir/2005/36/oj (Accessed January 1, 2024)

[11] EAEVE and FVE. (2023). European system of evaluation of veterinary training (ESEVT) – standard operating procedure (SOP) 2023. Available at: https://www.eaeve.org/fileadmin/downloads/SOP/ESEVT_SOP_2023_adopted_by_the_36th_GA_in_Leipzig_on_8_June_2023.pdf (Accessed December 28, 2023)

[12] Kanwischer M. Untersuchungen zu Digitalisierungsstrategien in der universitären Lehre zu Pandemiezeiten im Sommersemester 2020 an der Stiftung Tierärztliche Hochschule Hannover [Dissertation]. Hannover: Stiftung Tierärztliche Hochschule Hannover (n.d.).

[13] Lee DK. Alternatives to P value: confidence interval and effect size. Korean J Anesthesiol. (2016) 69:555–62. doi: 10.4097/kjae.2016.69.6.555, PMID: 27924194 PMC5133225

[14] Edwards P Roberts I Clarke M Diguiseppi C Wentz R Kwan I. Methods to increase response to postal and electronic questionnaires (review). Cochrane Database Syst. Rev. (2009) 2009:MR000008. doi: 10.1002/14651858.MR000008.pub4, PMID: 19588449 PMC8941848

[15] Jones TL Baxter MA Khanduja V. A quick guide to survey research. Ann R Coll Surg Engl. (2013) 95:5–7. doi: 10.1308/003588413X13511609956372, PMID: 23317709 PMC3964639

[16] Ehlers JP Voss B Kröger A Siegling-Vlitakis C Birk S Mülling C In: ., editor. KELDAT – a competence Centre of Veterinary Education Built by all German speaking veterinary universities. Bristol: VetED Symposium (2014). 30.

[17] Schaper E. eCompetence and Utilities for Learners and Teachers (eCULT+): Schlussbericht eCULT+: Förderzeitraum: 01.10.2016–28.02.2021. Hannover: Stiftung Tierärztliche Hochschule Hannover (2021).

[18] Schaper E Wissing S Kunzmann P Tipold A. Schlussbericht zu FERTHIK II (FERTHIK II) – Vermittlung von tiermedizinischen, klinischen Fertigkeiten und Implementierung von Ethik in der Tiermedizin. Hannover: Stiftung Tierärztliche Hochschule Hannover (2021).

[19] Fejzic N Serić-Haracić S Ayaz ND Meneghi D Abu-Basha E Tligui N. Experiences in delivering teaching and learning practices in establishments of veterinary education of the Mediterranean region under COVID-19 pandemic: From crisis to opportunities. Acta Vet Eurasia. (2022) 48:143–52. doi: 10.54614/actavet.2022.21102

[20] Naundorf H. Untersuchung der Hybridsemester-Lehre während der COVID-19-Pandemie an der Stiftung Tierärztliche Hochschule Hannover [Dissertation]. Hannover: Stiftung Tierärztliche Hochschule Hannover (2023).

[21] Bosse E Lübcke M Book A Würmseer G. (2020). Corona@Hochschule. Befragung von Hochschulleitungen zur (digitalen) Lehre.

[22] Hense J Goertz L. (2023). Monitor Digitalisierung 360° Arbeitspapier Nr. 67.

[23] Kerres M. (2022). Kerres, Michael (2022): Frustration in Videokonferenzen vermeiden. Grundlagen der Weiterbildung - Praxishilfen. Loseblattsammlung 7.50.160. Köln: Wolters Kluwer. [reprint].

[24] Hertling SF Back DA Eckhart N Kaiser M Graul I. How far has the digitization of medical teaching progressed in times of COVID-19? A multinational survey among medical students and lecturers in German-speaking Central Europe. BMC Med Educ. (2022) 22:387. doi: 10.1186/s12909-022-03470-z, PMID: 35596161 PMC9121080

[25] Naundorf H Tipold A Schaper E. Was nehmen wir mit für die Zukunft? – Befragung von Studierenden zum Tiermedizinstudium in COVID-19-Zeiten. Berl Munch Tierarztl Wochenschr. (2023) 136:1–17. doi: 10.2376/1439-0299-2022-19

[26] Herrmann-Werner A Erschens R Zipfel S Loda T. Medical education in times of COVID-19: survey on teachers' perspectives from a German medical faculty. GMS. J Med Educ. (2021) 38:1489. doi: 10.3205/zma001489, PMID: 34286073 PMC8256125

[27] Reinmann G Bohndick C Lübcke E Brase A Kaufmann M Groß N. (2020). "Emergency Remote Teaching im Sommersemester 2020" Bericht zur Begleitforschung – Lehrendenbefragung. Available at: https://www.hul.uni-hamburg.de/forschung/projektarchiv/ert/begleitforschung-bericht-2020-2.pdf

[28] Gomez O Garcia-Manzanares M Chicharro D Juarez M Llamazares-Martin C Soriano E. Application of blended learning to veterinary gross anatomy practical sessions: Students' perceptions of their learning experience and academic outcomes. Animals (Basel). (2023) 13:1666. doi: 10.3390/ani13101666, PMID: 37238097 PMC10215638

[29] Simons MC Pulliam D Hunt JA. The impact of the COVID-19 pandemic on veterinary clinical and professional skills teaching delivery and assessment format. J Vet Med Educ. (2022) 50:61–76. doi: 10.3138/jvme-2021-0106, PMID: 35038389

[30] Baillie S Decloedt A Frendo LM. Designing flipped classrooms to enhance learning in the clinical skills laboratory. J Vet Med Educ. (2022) 49:699–704. doi: 10.3138/jvme-2021-0043, PMID: 34369854

[31] Brombacher-Steiert S Ehrich R Schneider C Muller LR Tipold A Wissing S. Teaching clinical practical and communication skills of the clinical skills lab of the University of Veterinary Medicine Hannover, Foundation, Germany during the COVID-19 pandemic. GMS. J Med Educ. (2021) 38:1482. doi: 10.3205/zma001482, PMID: 34286066 PMC8256131

[32] Muehlberg J Tipold A Heppelmann M Wissing S. Simulator-assisted training of Abomasal surgery-a pilot study using blended learning and face-to-face teaching. Animals (Basel). (2023) 13:3822. doi: 10.3390/ani13243822, PMID: 38136859 PMC10740769

[33] Matthew SM Schoenfeld-Tacher RM Danielson JA Warman SM. Flipped classroom use in veterinary education: a multinational survey of faculty experiences. J Vet Med Educ. (2019) 46:97–107. doi: 10.3138/jvme.0517-058r1, PMID: 30418806

[34] Hamilton E Puschner B Olivier NB Gray C O’Connor A. Planning for next time: the challenges faced by a veterinary teaching hospital during the COVID-19 pandemic. J Vet Med Educ. (2023): e20220123. doi: 10.3138/jvme-2022-0123 39500504

[35] Gray K Tobin J. Introducing an online community into a clinical education setting: a pilot study of student and staff engagement and outcomes using blended learning. BMC Med Educ. (2010) 10:6. doi: 10.1186/1472-6920-10-6, PMID: 20100354 PMC2828452

[36] Liu Q Peng W Zhang F Hu R Li Y Yan W. The effectiveness of blended learning in health professions: systematic review and Meta-analysis. J Med Internet Res. (2016) 18:e2. doi: 10.2196/jmir.4807, PMID: 26729058 PMC4717286

[37] Dorrestein L Jansen J Plagis T Ritter C Vertenten G Barkema HW. Use of an online gaming tool, the veterinary DialogueTrainer, for teaching clinical communication skills to bovine veterinary practitioners. Front Vet Sci. (2023) 10:1192598. doi: 10.3389/fvets.2023.1192598, PMID: 37538168 PMC10394235

[38] Marahrens H Wagener MG Schaper E Zintl J Kiene F Ganter M. Teaching clinical hematology and leukocyte differentiation in veterinary medicine using virtual patients. Front Vet Sci. (2023) 10:1163927. doi: 10.3389/fvets.2023.1163927, PMID: 37795012 PMC10546049

[39] Kneissl SM Tichy A Mitlacher SF. Flipped classroom to facilitate deeper learning in veterinary undergraduate students: an educational change pilot study limited to the imaging module bones. Animals. (2023) 13:1540. doi: 10.3390/ani13091540, PMID: 37174577 PMC10177558

[40] Routh J Paramasivam SJ Cockcroft P Nadarajah VD Jeevaratnam K. Veterinary education during Covid-19 and beyond-challenges and mitigating approaches. Animals (Basel). (2021) 11:1818. doi: 10.3390/ani11061818, PMID: 34207202 PMC8234198

