# Supplementary material for: Veterinary teaching in COVID-19 times: perspectives of university teaching staff
Source: Front Vet Sci. 2024 Jun 27;11:1386978. doi: 10.3389/fvets.2024.1386978 (PMC11238364; doi:10.3389/fvets.2024.1386978)
Supplement: SUPPLEMENTARY DATA SHEET 1 — Excerpt from the online questionnaire about the digital summer semester 020 for lecturers and teaching staff at the University of Veterinary Medicine Hannover, Foundation with the questions evaluated in the article. [file Data_Sheet_1.PDF]

Dear professors,
Dear lecturers and teaching staff,

We would like to ask you to take part in this survey to evaluate the summer semester 2020.

The current COVID-19 situation has presented new challenges this summer semester. Face-to-face teaching was quickly switched to a digital semester.
In order to learn as much as possible from this time for future online teaching at the TiHo and thus further optimize it, your experiences and opinions are needed.

The survey will take about 25-30 minutes.

The survey was created as part of my doctoral thesis in the field of educational research and will be analyzed afterwards. Your data will be stored anonymously.
It is best to complete the survey on a computer/laptop, as the presentation is optimized for this.

Thank you for your support,

Miriam Kanwischer
Prof. Dr. Andrea Tipold, VPL

There are 27 questions in this survey.

General personal details

Which gender do you identify with? *

❗ Choose one of the following answers
Please choose **only one** of the following:

- ◯ Female
- ◯ Male
- ◯ Non-binary
- ◯ No answer

How old are you? *

❶ Choose one of the following answers
Please choose **only one** of the following:

- ◯ < 20
- ◯ 20
- ◯ 21
- ◯ 22
- ◯ 23
- ◯ 24
- ◯ 25
- ◯ 26
- ◯ 27
- ◯ 28
- ◯ 29
- ◯ 30
- ◯ 31
- ◯ 32
- ◯ 33
- ◯ 34
- ◯ 35
- ◯ 36
- ◯ 37
- ◯ 38
- ◯ 39
- ◯ 40
- ◯ 41
- ◯ 42
- ◯ 43
- ◯ 44
- ◯ 45
- ◯ 46
- ◯ 47
- ◯ 48
- ◯ 49
- ◯ 50
- ◯ 51
- ◯ 52
- ◯ 53
- ◯ 54
- ◯ 55
- ◯ 56
- ◯ 57
- ◯ 58
- ◯ 59
- ◯ 60
- ◯ 61

- 62
- 63
- 64
- 65
- 66
- 67
- 68
- 69
- 70
- 71
- 72
- 73
- 74
- 75
- 76
- 77
- 78
- 79
- 80
- > 80

Which respective subject area can your department be assigned to? *

Choose one of the following answers
Please choose **only one** of the following:

- Preliminary preclinical examination course (Vorphysikum)
- Intermediary preclinical examination course (Physikum)
- Clinical
- Paraclinical
- No answer

What position do you have at your institute? *

Choose one of the following answers
Please choose **only one** of the following:

- Institute/ Clinic director
- Working group leader
- Private lecturer
- Resident
- Assistant doctor
- Internship-Programm-Teilnehmende/r
- Research associates
- Other
- No answer

How long have you been actively involved in teaching? *

❶ Choose one of the following answers
Please choose **only one** of the following:

- < 1 year
- 1-5 years
- 6-10 years
- > 10 years
- No answer

Start of the digital semester

How often did you use the following digital courses as part of your teaching activities at the TiHo **BEFORE** the switch to the digital semester? *

Please choose the appropriate response for each item:

	Frequently	Often	Sometimes	Rarely	Never	No answer
**Virtual microscope**	○	○	○	○	○	○
**Recorded lectures**	○	○	○	○	○	○
**Recorded talks**	○	○	○	○	○	○
**Electronic teaching materials (e.g., PDF scripts)**	○	○	○	○	○	○
**Electronic textbooks (e.g., e-books)**	○	○	○	○	○	○
**CASUS case studies**	○	○	○	○	○	○
**TiHoMoodle course**	○	○	○	○	○	○
**YouTube channel (e.g., TiHoVideos)**	○	○	○	○	○	○
**Educational films**	○	○	○	○	○	○
**Online seminars**	○	○	○	○	○	○
**Social Networks (Facebook, NOVICE)**	○	○	○	○	○	○
**Self-tests (e.g., PTT)**	○	○	○	○	○	○
**Learning software**	○	○	○	○	○	○
**Podcasts**	○	○	○	○	○	○
**Heartsound Library**	○	○	○	○	○	○

What proved to be helpful in the **initial phase** of the semester? *

Please choose the appropriate response for each item:

	Extremly helpful	Very helpful	Not so helpful	Not at all helpful	I do not know the offer	No answer
**General information on the semester course via the TiHo homepage under "News and press" on "Measures regarding the coronavirus SARS-CoV-2 at the TiHo"**	◯	◯	◯	◯	◯	◯
**Advice, instructions, and technical support from Department 5 - Information and Data Processing Service (IDS)**	◯	◯	◯	◯	◯	◯
**Advice, instructions, and technical support from the Center for E-Learning, Didactics and Training Research**	◯	◯	◯	◯	◯	◯
**Information from the university management about the course schedule**	◯	◯	◯	◯	◯	◯
**Consultation with colleagues about the course schedule**	◯	◯	◯	◯	◯	◯
**Cooperation between lecturers and students**	◯	◯	◯	◯	◯	◯
**Flexibility of students with regard to changing the course**	◯	◯	◯	◯	◯	◯

What caused you difficulties in the **initial phase** of the semester? Please give your assessment of the following points. *

Please choose the appropriate response for each item:

	Strongly agree	Agree	Disagree	Strongly disagree	No answer
**Getting an overview of the available digital systems**	○	○	○	○	○
**Organizing digitization of teaching materials**	○	○	○	○	○
**Digitization of theoretical content**	○	○	○	○	○
**Digitization of practical content**	○	○	○	○	○
**Own time management**	○	○	○	○	○
**Own equipment with internet-enabled devices and additional equipment (headset, camera)**	○	○	○	○	○
**Coordination between colleagues at own institute**	○	○	○	○	○
**Coordination with colleagues at other institutes**	○	○	○	○	○
**Personal circumstances**	○	○	○	○	○

Technical requirements

Please indicate how often you use the following internet-enabled devices for teaching in the digital semester. *

Please choose the appropriate response for each item:

	Frequently	Often	Sometimes	Rarely	Never	No answer
**Notebook/Laptop**	○	○	○	○	○	○
**Smartphone**	○	○	○	○	○	○
**Tablet**	○	○	○	○	○	○
**Desktop PC**	○	○	○	○	○	○

What additional technical equipment do you have? (multiple choice possible) *

❶ Check all that apply

Please choose **all** that apply:

- [ ] Headphones
- [ ] Headset
- [ ] Webcam
- [ ] Microphone
- [ ] Speakers or integrated loudspeaker systems
- [ ] Printer
- [ ] Scanner
- [ ] External storage media
- [ ] No answer
- [ ] Other: ____________

Teaching in the digital semester

Please rate the following statements. *

Please choose the appropriate response for each item:

	Strongly agree	Agree	Disagree	Strongly disagree	No answer
**It was clear to me during the semester how the digital semester would work.**	◯	◯	◯	◯	◯
**It is clear to me how things will continue until the end of the summer semester.**	◯	◯	◯	◯	◯
**I communicated to the students how the digital semester would work.**	◯	◯	◯	◯	◯
**Digital competence in the team was conducive to implementing innovative solutions for digital teaching.**	◯	◯	◯	◯	◯
**I knew which platforms were available for my type of teaching.**	◯	◯	◯	◯	◯
**I always had contacts/support to help me digitize my teaching content in a meaningful way.**	◯	◯	◯	◯	◯
**The instructions and information material on how to digitize my teaching content were helpful.**	◯	◯	◯	◯	◯
**I was able to quickly become used to new systems.**	◯	◯	◯	◯	◯
**My previous experience with similar systems helped me with the implementation of digital teaching.**	◯	◯	◯	◯	◯
**Digital teaching was able to meet my quality standards.**	◯	◯	◯	◯	◯
**Digital teaching enabled me to offer interesting additional material for which there would otherwise have been no space.**	◯	◯	◯	◯	◯
**I had the feeling that the students were well versed in using the technology.**	◯	◯	◯	◯	◯
**During synchronous teaching, I had the opportunity to get feedback from the participants.**	◯	◯	◯	◯	◯
**The collaboration with the students was constructive.**	◯	◯	◯	◯	◯
**The students understood the situation and reacted flexibly.**	◯	◯	◯	◯	◯

Digital teaching currently takes place both synchronously in the form of live lectures and live question and answer sessions and asynchronously in the form of digital learning material, assignments, and learning control units. What is the best way for you to teach?

*

❗ Choose one of the following answers

Please choose **only one** of the following:

- ◯ Synchronous teaching
- ◯ Asynchronous teaching
- ◯ Combination of synchronous and asynchronous teaching
- ◯ I don't know

How often did you use the following digital teaching services as part of your teaching activities at the TiHo **DURING** the digital semester? *

Please choose the appropriate response for each item:

	Frequently	Often	Sometimes	Rarely	Never	No answer
**Digital live events**	◯	◯	◯	◯	◯	◯
**Digital live work in small groups**	◯	◯	◯	◯	◯	◯
**Digital live Q&A sessions**	◯	◯	◯	◯	◯	◯
**Video of the lecturer with view of the presentation including audio track**	◯	◯	◯	◯	◯	◯
**Video of the presentation with audio track**	◯	◯	◯	◯	◯	◯
**Audio files/podcast with accompanying PDF script**	◯	◯	◯	◯	◯	◯
**Audio files/podcast without PDF script**	◯	◯	◯	◯	◯	◯
**Instructional videos on practical exercises**	◯	◯	◯	◯	◯	◯
**Instructional videos on theoretical content**	◯	◯	◯	◯	◯	◯
**Tasks with digital submission**	◯	◯	◯	◯	◯	◯
**Learning control questions (as test performance)**	◯	◯	◯	◯	◯	◯
**Learning control questions/ quizzes (for self-monitoring)**	◯	◯	◯	◯	◯	◯
**Digital (clinical) learning cases**	◯	◯	◯	◯	◯	◯
**PDF scripts with text commentary**	◯	◯	◯	◯	◯	◯
**PDF scripts without text commentary**	◯	◯	◯	◯	◯	◯
**Guided forum/ forum posts**	◯	◯	◯	◯	◯	◯
**Digital accompanying material (e-book chapters, articles from journals)**	◯	◯	◯	◯	◯	◯
**Instructions for practical exercises at home**	◯	◯	◯	◯	◯	◯

In your opinion, what measures help students to process digital material promptly? *

Please choose the appropriate response for each item:

	Strongly agree	Agree	Disagree	Strongly disagree	No answer
**Learning instructions**	○	○	○	○	○
**Compulsory courses**	○	○	○	○	○
**Deadlines**	○	○	○	○	○
**Assignment tasks**	○	○	○	○	○
**Learning control questions/ quizzes**	○	○	○	○	○
**Pretests (requirements to be able to view further content)**	○	○	○	○	○
**Course attendance certificate**	○	○	○	○	○
**Q&A sessions (e.g., via Microsoft Teams)**	○	○	○	○	○
**Exchange possibilities in the forum**	○	○	○	○	○
**Progress indicator at TiHoMoodle (blue check mark)**	○	○	○	○	○

How should lectures be recorded? *

❗ Choose one of the following answers

Please choose **only one** of the following:

- ○ As a whole
- ○ In chapters
- ○ No answer

When a lecture recording is divided into individual chapters, how long should the individual chapters be? *

Only answer this question if the following conditions are met:
Answer was 'In chapters' at question '15 [e10]' (How should lectures be recorded?)

❶ Choose one of the following answers
Please choose **only one** of the following:

- ◯ 0-10 minutes
- ◯ 11-20 minutes
- ◯ 21-30 minutes
- ◯ 31-40 minutes
- ◯ 41-50 minutes
- ◯ 51-60 minutes
- ◯ 61-70 minutes
- ◯ 71-80 minutes
- ◯ 81-90 minutes
- ◯ No answer

Effects of the digital semester on teaching

Has the switch to a digital semester generally had a negative impact on you? *

❶ Choose one of the following answers
Please choose **only one** of the following:

- ◯ Yes
- ◯ No
- ◯ No answer

How does the time required for your digital teaching compare to conventional face-to-face courses with teaching materials (e.g. scripts)? *

❶ Choose one of the following answers
Please choose **only one** of the following:

- ◯ The effort is higher
- ◯ The effort is the same
- ◯ The effort is lower
- ◯ No answer

Please evaluate the following statements on the learning behavior and active participation of students in the digital semester. *

Please choose the appropriate response for each item:

	Strongly agree	Agree	Disagree	Strongly disagree	No answer
**Students were less reserved about asking questions.**	◯	◯	◯	◯	◯
**The students participated more actively in the digital events compared to face-to-face events.**	◯	◯	◯	◯	◯
**Students only took part in courses that were compulsory.**	◯	◯	◯	◯	◯
**More students took part in courses that were not compulsory compared to the analog semester.**	◯	◯	◯	◯	◯

Please rate the following statements about your flexibility | self-organization | resilience in the digital semester. *

Please choose the appropriate response for each item:

	Strongly agree	Agree	Disagree	Strongly disagree	No answer
**The digital semester allows me to be more flexible in my teaching.**	○	○	○	○	○
**The increased flexibility improves my everyday life.**	○	○	○	○	○
**I think the increased flexibility is positive in general.**	○	○	○	○	○
**I have more time.**	○	○	○	○	○
**I have worked at other times of the day.**	○	○	○	○	○
**Synchronous teaching has not changed my way of teaching.**	○	○	○	○	○
**Synchronous teaching has not changed my daily routine.**	○	○	○	○	○
**Asynchronous teaching has not changed my way of teaching.**	○	○	○	○	○
**Asynchronous teaching has not changed my daily routine.**	○	○	○	○	○
**I've reorganized my teaching because of the digital semester.**	○	○	○	○	○
**Due to the digital semester, my teaching has become more problem-oriented.**	○	○	○	○	○
**The intensive work at the computer screen has challenged me.**	○	○	○	○	○
**I was able to digitize my teaching content using existing hardware (PC, notebook).**	○	○	○	○	○
**I have a workstation at my disposal where I can concentrate on my work.**	○	○	○	○	○
**I feel less stressed overall.**	○	○	○	○	○
**I like the fact that I can make lecture recordings and videos available for revision.**	○	○	○	○	○
**I see the digital semester as a personal challenge.**	○	○	○	○	○
**I miss the direct contact with the students.**	○	○	○	○	○
**I find it difficult to teach with digital teaching materials.**	○	○	○	○	○

	Strongly agree	Agree	Disagree	Strongly disagree	No answer
**I'll be happy when my course is back to how it was before Corona.**	◯	◯	◯	◯	◯
**I have not developed a routine for the online semester.**	◯	◯	◯	◯	◯
**I had difficulties organizing private care tasks (e.g. childcare, caring for relatives).**	◯	◯	◯	◯	◯

Have you encountered any other challenges this semester? *

❶ Choose one of the following answers
Please choose **only one** of the following:

◯ No
◯ No answer
◯ Other

Outlook and general suggestions

Where do you see room for improvement with regard to digital teaching? *

Please choose the appropriate response for each item:

	Strongly agree	Agree	Disagree	Strongly disagree	No answer
**Technical equipment at the TiHo**	◯	◯	◯	◯	◯
**Own technical equipment**	◯	◯	◯	◯	◯
**Training of lecturers on online teaching**	◯	◯	◯	◯	◯
**Training of students on online teaching**	◯	◯	◯	◯	◯
**Findability of instructions**	◯	◯	◯	◯	◯
**More interactive courses**	◯	◯	◯	◯	◯

Would you welcome the sustainability of the following systems (after coronavirus)?", *

Please choose the appropriate response for each item:

	Strongly agree	Agree	Disagree	Strongly disagree	No answer
**Microsoft Teams**	◯	◯	◯	◯	◯
**TiHoMoodle**	◯	◯	◯	◯	◯

Which teaching method do you see the greatest potential in the future? *

Please choose the appropriate response for each item:

	Strongly agree	Agree	Disagree	Strongly disagree	No answer
**Digital live events, e.g., via Microsoft Teams (synchronous)**	◯	◯	◯	◯	◯
**Blended learning***	◯	◯	◯	◯	◯
**Flipped/inverted classroom****	◯	◯	◯	◯	◯
**Asynchronous online teaching (e.g., presentation recordings, exercises in TiHoMoodle, teaching videos)**	◯	◯	◯	◯	◯
**Both synchronous and asynchronous online teaching**	◯	◯	◯	◯	◯

* Combination of classroom teaching and online units.

** Combination of initial preparation of learning content (e.g., watching lecture recordings), and subsequent discussion of questions.

What did you particularly like in this digital semester?

Please write your answer here:

Do you have any suggestions for improving digital teaching?

Please write your answer here:

What technology/software did you miss for teaching digital courses in the summer semester 2020?

Please write your answer here:

Thank you for taking the time to answer this questionnaire!

Contact for further questions:

Miriam Kanwischer

ZELDA -E-Learning Service

Bünteweg 11, 30559 Hanover

Miriam.Kanwischer@tiho-hannover.de

08-14-2020 – 10:06

Submit your survey.
Thank you for completing this survey.
