# Supplementary figures and images for: Veterinary teaching in COVID-19 times: perspectives of university teaching staff
Source: Front Vet Sci. 2024 Jun 27;11:1386978. doi: 10.3389/fvets.2024.1386978 (PMC11238364; doi:10.3389/fvets.2024.1386978)

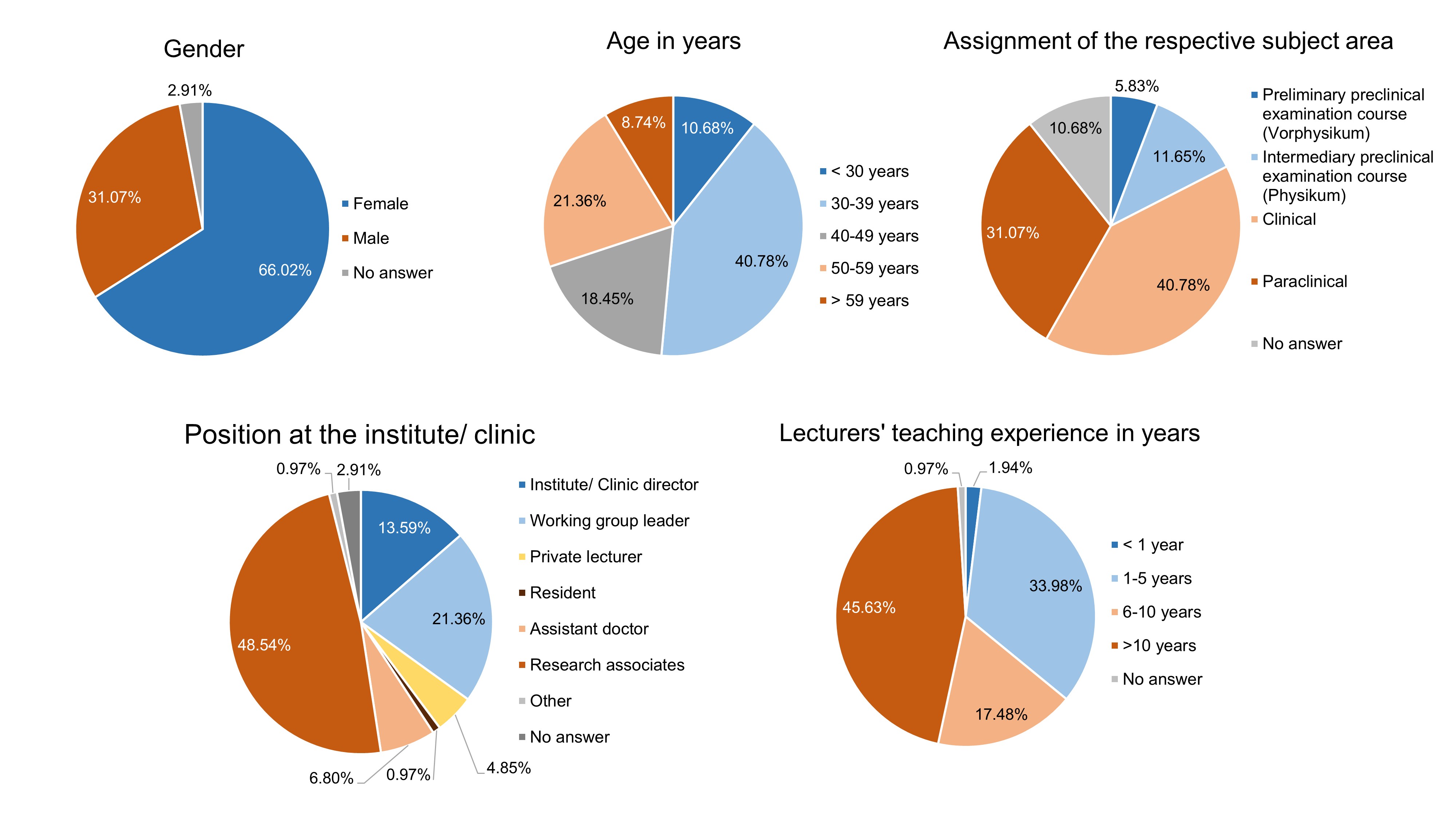

Supplement: SUPPLEMENTARY FIGURE 1 — Online survey of teaching staff at the University of Veterinary Medicine Hannover, Foundation. Graphical representation of the general personal data (n = 103). [file Image_1.JPEG]
